# Small Molecule Ice Recrystallization Inhibitors Enable Freezing of Human Red Blood Cells with Reduced Glycerol Concentrations

**DOI:** 10.1038/srep09692

**Published:** 2015-04-08

**Authors:** Chantelle J. Capicciotti, Jayme D. R. Kurach, Tracey R. Turner, Ross S. Mancini, Jason P. Acker, Robert N. Ben

**Affiliations:** 1 Department of Chemistry, D'Iorio Hall, 10 Marie Curie, University of Ottawa, Ottawa, ON, Canada, K1N 6N5; 2 Canadian Blood Services, Centre for Innovation, 8249 – 114 Street NW, Edmonton, AB, Canada, T6G 2R8

## Abstract

In North America, red blood cells (RBCs) are cryopreserved in a clinical setting using high glycerol concentrations (40% w/v) with slow cooling rates (~1°C/min) prior to storage at −80°C, while European protocols use reduced glycerol concentrations with rapid freezing rates. After thawing and prior to transfusion, glycerol must be removed to avoid intravascular hemolysis. This is a time consuming process requiring specialized equipment. Small molecule ice recrystallization inhibitors (IRIs) such as β-PMP-Glc and β-pBrPh-Glc have the ability to prevent ice recrystallization, a process that contributes to cellular injury and decreased cell viability after cryopreservation. Herein, we report that addition of 110 mM β-PMP-Glc or 30 mM β-pBrPh-Glc to a 15% glycerol solution increases post-thaw RBC integrity by 30-50% using slow cooling rates and emphasize the potential of small molecule IRIs for the preservation of cells.

Red blood cell (RBC) transfusions are lifesaving because they increase RBC mass in patients with low oxygen-carrying capacity resulting from acute blood loss (traumatic/surgical haemorrhage), decreased RBC production in bone marrow (leukemias), or decreased RBC survival (hemolytic anemias)^1^. Hypothermic preservation of RBCs has enabled blood banks to ensure a readily available and safe blood supply. However, hypothermic storage is limited to 42 days and this can result in shortages of blood when high numbers of emergency blood transfusions are required such as in the wake of a natural disaster^2^,^3^,^4^. Cryopreservation of RBC units can extend storage time to 10 years, however this preservation method is largely limited to rare blood phenotypes, autologous RBC storage and military applications for blood storage^1^,^5^,^6^.

One of the main reasons cryopreserved RBC units are not routinely used in transfusion medicine is that current cryopreservation protocols do not permit the direct transfusion of RBCs immediately after thawing. In Canada and the United States, clinical RBC cryopreservation protocols utilize high concentrations of the cell permeating cryoprotectant glycerol (>40% wt/vol) with slow cooling rates (1°C/minute) and storage at −80°C in a mechanical freezer to ensure high recovery of RBCs^1^,^7^. However, complicated and time consuming post-thaw deglycerolization procedures are necessary to ensure glycerol concentrations are reduced to less than 1% prior to transfusion to prevent post-transfusion intravascular hemolysis^5^,^7^,^8^,^9^. Furthermore, these units must be transfused within 24 hours imposing logistical challenges due to significant time and/or distance constraints on the transportation of thawed RBCs^10^,^11^. While current cryopreservation methods are less than ideal for large-scale inventory management^5^, it is the only technology that can readily deliver large quantities of pre-screened RBC units to patients^12^. Thus, improved cryopreservation methods using non-toxic cryoprotective additives with significantly reduced amounts of glycerol to decrease post-thaw processing times are required.

Ice recrystallization during freezing and thawing is a significant contributor to cellular damage and death^13^,^14^,^15^. As a consequence, there has been tremendous interest in designing compounds that can inhibit ice recrystallization for cryopreservation^16^,^17^,^18^,^19^,^20^,^21^. Our laboratory has recently reported the rational design and synthesis of large *C*-linked glycoconjugates^22^,^23^,^24^ as well as small molecules^25^,^26^,^27^ that inhibit the process of ice recrystallization. We hypothesize that inhibiting this process will greatly reduce cellular injury during cryopreservation and increase the post-thaw recovery of cells with specialized cellular functions^24^,^28^. Current research on improving RBC cryopreservation has focused on a number of different aspects of the cryopreservation protocol in an attempt to overcome the limitations associated with using glycerol as a cryoprotectant. Towards this end, other cryoprotectants have been examined including non-penetrating additives such as hydroxyethyl starch (HES), polyvinylpyrrolidone (PVP) and dextran^1^,^29^,^30^,^31^,^32^. In a recent report, polyvinyl alcohol (PVA) has been shown to inhibit ice recrystallization and when used in combination with HES can cryopreserve ovine and human RBCs with rapid cooling rates^33^. Simple carbohydrates and oligosaccharides such as trehalose have also been explored as cryoprotectants for RBCs^34^,^35^,^36^,^37^. To date, the use of small molecule ice recrystallization inhibitors for the cryopreservation of human RBCs has not been explored.

In this paper we report several novel small molecule phenolic-glycosides that are inhibitors of ice recrystallization. These compounds are also effective cryo-additives capable of decreasing the amount of post-thaw hemolysis of human RBCs after freezing in the presence of reduced glycerol concentrations with slow freezing rates. This is the first example of a small molecule inhibitor of ice recrystallization effectively freezing RBCs with reduced amounts of glycerol and slow freezing rates. These results have tremendous potential for improving the cryopreservation of RBCs as post-thaw deglycerolization processing times would be greatly reduced with low glycerol concentrations.

## Results

### IRI activity of para-methoxyphenyl-β-d-glycosides

**Figure 1:**
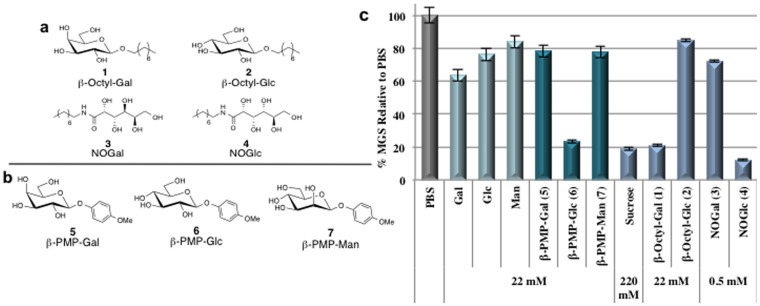
Structures and IRI activity of small carbohydrate-based molecules. (a) Structures of the first low molecular weight carbohydrate-based ice recrystallization inhibitors^25^. (b) Structures of PMP containing phenolic-glycosides β-PMP-Gal (**5**), β-PMP-Glc, (**6**) and β-PMP-Man (**7**). (c) IRI activity of β-PMP-Gal (**5**), β-PMP-Glc (**6**), and β-PMP-Man (**7**) at 22 mM compared with sucrose (at 220 mM)^28^, β-Octyl-Gal and –Glc (**1**–**2** at 22 mM), and NOGal and NOGlc (**3**–**4** at 0.5 mM)^25^. All compounds are represented as a % MGS (mean grain size) of ice crystals (n = 108) relative to the PBS positive control. Error bars represent the standard error of the mean (SEM).

Prior work by our laboratory has demonstrated that amphiphilic small molecule carbohydrate derivatives (Fig. 1a) as well as some amino acid derivatives are very effective inhibitors of ice recrystallization^25^,^26^,^27^. In all of these compounds, the hydrophobic component of the molecule was an alkyl chain varying from 6–14 carbons in length. While these molecules exhibited potent ice recrystallization inhibition (IRI) activity, they also possessed surfactant-like properties that are detrimental to cell membranes^38^,^39^,^40^. Generally speaking, this precludes the use of these compounds in cells. In the present study we chose to employ an aryl ring as a hydrophobic moiety instead of an alkyl chain. Consequently, phenolic-glycosides of d-galactose, d-glucose and d-mannose bearing a β-linked *para*-methoxyphenyl (PMP) moiety (Fig. 1b) were synthesized (supplementary information) and assessed for their ability to inhibit ice recrystallization^41^,^42^. IRI activity is represented as a ratio of the mean ice crystal grain size in a given sample to the mean ice crystal size of phosphate buffered saline (PBS) solution, a positive control for ice recrystallization. Thus, smaller mean grain size (MGS) values are indicative of smaller ice crystal sizes and hence greater IRI activity (Fig. 1).

d-Galactose derivative β-PMP-Gal (**5**) and d-mannose derivative β-PMP-Man (**7**) both exhibited weak IRI activity that was similar to that exhibited by d-mannose (Fig. 1c). However, d-glucose derivative β-PMP-Glc (**6**) was a very effective inhibitor of ice recrystallization and was significantly more active than β-PMP-Gal (**5**) and β-PMP-Man (**7**). In fact, β-PMP-Glc (**6**) exhibited activity similar to the previously reported non-ionic surfactant β-Octyl-Gal (**1**) at equimolar concentration^25^. β-PMP-Glc (**6**) also exhibited comparable IRI activity to a 220 mM solution of sucrose, which is a common ingredient of many cryopreservation solutions^28^.

Our laboratory has previously correlated IRI activity to carbohydrate hydration and reported that glycoconjugates containing highly hydrated monosaccharides (such as d-galactose) were more IRI active than those containing less hydrated monosaccharides (d-glucose and d-mannose)^23^,^43^. This trend was also observed in small molecules where the non-ionic pyranose-based surfactants containing a highly hydrated d-galactose head group (β-Octyl-Gal, **1**, Fig. 1) was significantly more active than the surfactants containing the less hydrated d-glucose head group (β-Octyl-Glc, **2**)^25^. The fact that the d-glucopyranose derivative β-PMP-Glc (**6**) exhibited potent IRI activity and was significantly more IRI active than d-galactopyranose derivative β-PMP-Gal (**5**) (Fig. 1c) is unprecedented. This is the first example where a glucopyranose derivative is significantly more IRI active than a galactopyranose derivative. It was also surprising that both β-PMP-Gal (**5**) and β-PMP-Man (**7**) exhibited identical IRI activities despite the difference in hydration between these two pyranose residues^23^,^43^,^44^. Unfortunately, hydration numbers or molar compressibilities of **5**–**7** have not been reported in the literature, thus a correlation between the hydration characteristics of these compounds and their IRI activity is not possible at this time. However, a plausible explanation for these results is that the hydration characteristics of **5**–**7** are very different than the parent monosaccharides due to the presence of the PMP moiety.

### Freezing of red blood cells (RBCs) with IRI active small molecules

The freezing of human RBCs was carried out using a two-step graded freezing protocol to assess the effectiveness of small molecule IRIs as cryo-additives^34^,^45^,^46^. In this protocol, RBCs suspended in the cryoprotectant solution were slowly cooled at a rate of 1°C/min to pre-defined sub-zero temperatures and then rapidly cooled to −196°C (plunged into liquid nitrogen) prior to thawing. Following fast thawing, post-thaw hemolysis was quantified utilizing Drabkin's assay^47^,^48^. The two-step interrupted freezing protocol provides valuable information with respect to the mechanism of cryoinjury, and insight as to how a cryoprotectant is functioning^45^. RBC freezing results are reported as the percent post-thaw RBC integrity (100 - % hemolysis).

**Figure 2:**
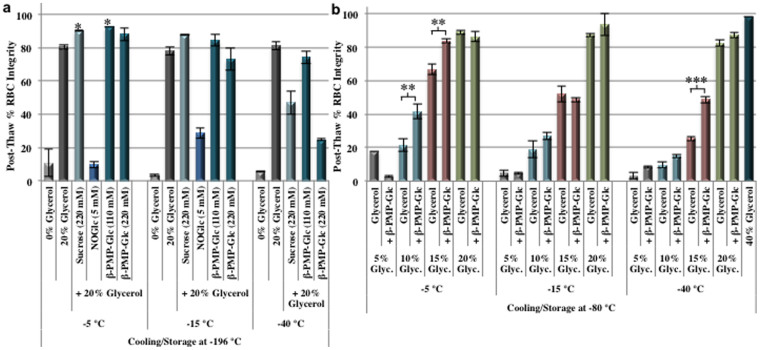
RBC freezing with novel small molecule IRIs. (a) The effect of NOGlc (**4**), β-PMP-Glc (**6**) and sucrose on post-thaw RBC integrities (n = 2). RBC samples in indicated cryo-solutions were slow-cooled 1°C/min to defined sub-zero temperatures and then rapidly cooled (cooling rate approximately 115°C/min) by immersion into liquid nitrogen and stored at −196°C. (b) The effect of 110 mM β-PMP-Glc (**6**) on post-thaw RBC integrity using reduced glycerol concentrations (n = 4–30). RBC samples in glycerol solutions with or without 110 mM β-PMP-Glc (**6**) were slowly cooled (1°C/min) to defined sub-zero temperatures and placed into dry ice and stored at −80°C. All samples were quickly thawed at 37°C and post-thaw hemolysis was quantified post-thaw using Drabkin's assay to determine % RBC integrity (100 - % hemolysis). Error bars represent SEM. Asterisks indicate statistical significance relative to the glycerol control as determined by two-tailed unpaired student's T-test: *, *p* < 0.05; **, *p* < 0.01; ***, *p* < 0.001.

Two different IRI active small molecules containing glucose, NOGlc (**4**) and β-PMP-Glc (**6**), were assessed for their ability to protect human RBCs from cryoinjury during freezing (Fig. 2). Previous work conducted by our laboratory has shown that simple carbohydrates were more effective cryoprotectants at concentrations approximately ten times greater than the concentration at which they are IRI active^28^. Thus, NOGlc (**4**), which is highly IRI active at 0.5 mM (Fig. 1c)^25^ was used in cryo-solutions at 2 mM or 5 mM concentrations while β-PMP-Glc (**6**) was used at 110 mM or 220 mM. NOGlc could not be assessed in the freezing assays at concentrations greater than 5 mM due poor solubility in aqueous solutions containing glycerol^25^,^49^. Sucrose was examined at concentrations of 220 mM and 500 mM as it has previously been identified as an effective inhibitor of ice recrystallization and can effectively cryopreserve human embryonic liver cells^28^. Solutions containing RBCs were cooled slowly at a rate of 1°C/min to −5, −15 or −40°C and then immersed in liquid nitrogen (−196°C) before being thawed.

Sucrose and NOGlc (**4**) were not effective cryo-additives for the freezing of RBCs (Fig. 2a, Supplementary Fig. S1). NOGlc in a 20% glycerol solution resulted in significant post-thaw RBC hemolysis (approximately 30% RBC integrity) upon slow cooling to −15°C. In contrast, post-thaw integrity with only 20% glycerol was 80%. Sucrose solutions (220 mM and 500 mM) in 20% glycerol resulted in post-thaw integrities of 85–90% when samples were immersed in liquid nitrogen (cooling rate of 115°C/min) from −5 or −15°C (Fig. 2a, Supplementary Fig. S1). However, RBC integrities were reduced to 47% when the samples were cooled slowly (cooling rate of 1°C/min) to −40°C and then transferred directly to −196°C. It is important to note that without the addition of glycerol to the cryoprotective solutions, the compounds did not prevent hemolysis resulting in post-thaw RBC integrity equal to that obtained with a dextrose/saline solution (Supplementary Fig. S1).

Interestingly, a 110 mM solution of small molecule ice recrystallization inhibitor β-PMP-Glc (**6**) in 20% glycerol resulted in slightly higher post-thaw RBC integrity than the 20% glycerol control. Under these conditions the sample was slowly cooled (1°C/min) to −5°C or −15°C and directly immersed into liquid nitrogen and stored at −196°C (Fig. 2a). Post-thaw RBC integrity with slow cooling to −5°C was 93% when the solution contained 110 mM β-PMP-Glc and 81% for 20% glycerol control. The 20% glycerol solutions had identical post-thaw integrities (75–80%) when slowly cooled to −40°C irrespective of whether 110 mM β-PMP-Glc was added. However, post-thaw RBC integrity was decreased to 25% when 220 mM β-PMP-Glc was used with the same freezing conditions. In contrast, the 220 mM sucrose solution with 20% glycerol resulted in only 48% post-thaw integrity. Overall, the results obtained with 110 mM β-PMP-Glc (**6**) were encouraging and suggested that this compound may be a beneficial additive for the freezing of RBCs using greatly reduced amounts of glycerol.

The 20% glycerol control is a very effective additive for the cryopreservation of RBCs and this concentration of glycerol is used in European countries in combination with rapid freezing rates (>100°C/min) followed by storage at −196°C^1^,^5^,^50^,^51^. In this protocol, fast freezing rates are essential in order to ensure adequate post-thaw RBC recoveries. However, in Canada and the United States, clinical cryopreservation protocols use higher concentrations of glycerol (typically 40%) with a slow rate-controlled freezing (1°C/min) to −80°C and subsequent storage at −80°C^1^,^4^,^8^,^52^. Consequently, the increase in post-thaw RBC recoveries observed with the 110 mM β-PMP-Glc solution containing only 20% glycerol using low freezing rates was very promising. Given the potential benefits to using reduced glycerol concentrations in the clinical setting (i.e. decreased post-thaw deglycerolization processing times), we investigated the ability of β-PMP-Glc to freeze RBCs to −80°C with reduced concentrations of glycerol.

Solutions containing 110 mM β-PMP-Glc with 5, 10, 15 or 20% glycerol were used to freeze human RBCs (Fig. 2b)^34^,^45^,^46^. Samples were cooled slowly at a rate of 1°C/min to the indicated sub-zero temperature and then placed directly into dry ice (−80°C). A slow cooling rate of 1°C/min and storage at −80°C was selected as these conditions closely reflect clinical RBC cryopreservation protocols utilized in North America. Under these conditions, low post-thaw RBC integrity was observed with 5% and 10% glycerol solutions (Fig. 2b). However, upon the addition of 110 mM β-PMP-Glc (**6**) to the 10% glycerol solution, post-thaw RBC integrity increased to 42% relative to 21% observed with 10% glycerol when samples were held at −5°C prior to cooling and storage at −80°C. The advantage of adding 110 mM β-PMP-Glc (**6**) is most evident with 15% glycerol where cooling to −5°C followed by immersion of the sample into dry ice (−80°C) increased RBC integrity to 84% from 67% with the 15% glycerol control. When RBCs were cooled to −15°C prior to immersion in dry ice, post-thaw RBC integrity decreased overall, but similar integrities were observed with the 15% glycerol solutions with or without β-PMP-Glc. However, β-PMP-Glc doubled the post-thaw RBC integrity when the sample was slowly cooled to −40°C prior to storage at −80°C (49% with β-PMP-Glc versus 25% for 15% glycerol alone).

**Figure 3:**
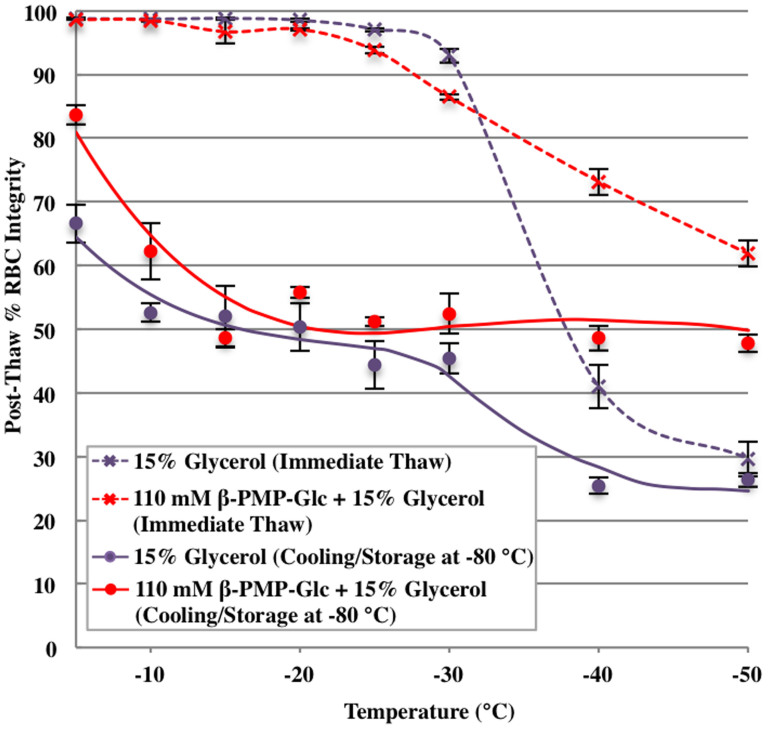
Two-step cooling profile of β-PMP-Glc. The effect of 15% glycerol solutions on post-thaw RBC integrity (100 - % hemolysis) with and without 110 mM β-PMP-Glc (**6**). Samples were slowly cooled (1°C/min) to pre-defined sub-zero temperatures then either immediately thawed (“immediate thaw” curves, dashed lines) or rapidly cooled and stored at −80°C prior to thawing at 37°C (“cooling/storage” curves, solid lines). Results are presented as mean post-thaw RBC integrity (n = 4–30). Error bars indicate standard error of the mean (SEM).

With the exciting result RBCs could be frozen using slow cooling rates with β-PMP-Glc and 15% glycerol, we sought to understand if this compound was protecting RBCs during the slow cooling step or during the rapid cooling step of the freezing protocol. Towards this end, we employed a two-step interrupted freezing protocol where RBC integrity was analysed at both stages, thus providing valuable information about the mechanism of cryoinjury in the presence of β-PMP-Glc (**6**)^45^. RBC samples were suspended in 15% glycerol solution with and without 110 mM β-PMP-Glc and slowly cooled (1°C/min) to sub-zero temperatures between −5 to −50°C. The samples were either thawed immediately (see dashed lines in Fig. 3) or rapidly cooled and stored at −80°C prior to thawing (see solid lines in Fig. 3)^46^.

The “cooling” profile for the 15% glycerol control (Fig. 3), where RBCs were thawed immediately after slow cooling to a defined sub-zero temperature is typical of a cell penetrating cryoprotectant where a progressive decrease in post-thaw RBC integrity is observed with lower sub-zero temperatures^46^ (purple dashed line). The decrease in post-thaw RBC integrity observed upon slow cooling to lower sub-zero temperatures (i.e. below −30°C) is a result of cellular damage due to concentrating solutes, consistent with Mazur's two-factor hypothesis of freezing injury^53^. However, high RBC post-thaw integrities were observed with slow freezing to temperatures above −30°C (Fig. 3). This is presumably due to the colligative effects of glycerol and its ability to decrease the amount of ice present at any sub-zero temperature, thus mitigating osmotic pressures across the cell membrane that ultimately result in cellular damage^46^,^53^. When β-PMP-Glc (110 mM) is added to the 15% glycerol solution, we observe a progressive increase in RBC integrity upon cooling to the lower sub-zero temperatures (red dashed line, Fig 3). For instance at −40°C, RBC integrity with 15% glycerol is 41% and this is increased to 73% with the addition of 110 mM β-PMP-Glc. Similarly, at −50°C post-thaw integrity with β-PMP-Glc was 62% compared to 29% for the 15% glycerol control. The fact that addition of β-PMP-Glc to a 15% glycerol solution resulted in less hemolysis is consistent with the profile of a cell penetrating cryoprotectant^46^, and suggests that β-PMP-Glc may be interacting with the RBC membrane and may even be internalized. These results clearly demonstrate that β-PMP-Glc provides additional protection during the slow-cooling phase of the freezing protocol when added to a 15% glycerol solution.

When samples were rapidly cooled to −80°C after slow cooling to sub-zero temperatures, different profiles were obtained (Fig. 3, solid lines) compared to the samples that were thawed immediately. Freezing of RBCs using 15% glycerol resulted in drastically reduced cell integrities even at higher sub-zero temperatures. When the samples were slowly cooled to temperatures lower than −30°C, RBC integrity was approximately 25% (purple solid lines, Fig. 3). Higher post-thaw integrities observed at temperatures above −30°C is attributed to the rapid cooling being initiated at higher sub-zero temperatures thus creating an overall faster cooling rate. These results are consistent with those reported elsewhere in the literature using fast cooling rates in conjunction with lower concentrations of glycerol^50^,^54^,^55^. When 110 mM β-PMP-Glc is added to RBCs in 15% glycerol, post-thaw RBC integrities are 5–15% better at all sub-zero cooling temperatures higher than −30°C (red solid lines, Fig. 3). Interestingly, β-PMP-Glc appears to have the greatest affect when the sample is slowly cooled to −40 or −50°C prior to rapid cooling to −80°C. The post-thaw RBC integrities are approximately 50% when the solution contains β-PMP-Glc, which is double that of the 15% glycerol solution alone.

Collectively, the results from the two-step interrupted freezing protocol experiments verify that β-PMP-Glc (**6**) can significantly decrease post-thaw RBC hemolysis when added to a 15% glycerol solution using slow cooling rates. β-PMP-Glc confers protection during both the slow-cooling and rapid cooling/storage at −80°C stages of the freezing protocol, regardless of the initial sub-zero cooling temperature. However, the greatest effect with β-PMP-Glc is observed at lower sub-zero temperatures (−40 or −50°C), which is significant as these freezing conditions mimic RBC clinical cryopreservation protocols utilizing slow freezing rates but higher glycerol concentrations (i.e. 40% glycerol).

### Pre-incubation of β-PMP-Glc with RBCs

**Figure 4:**
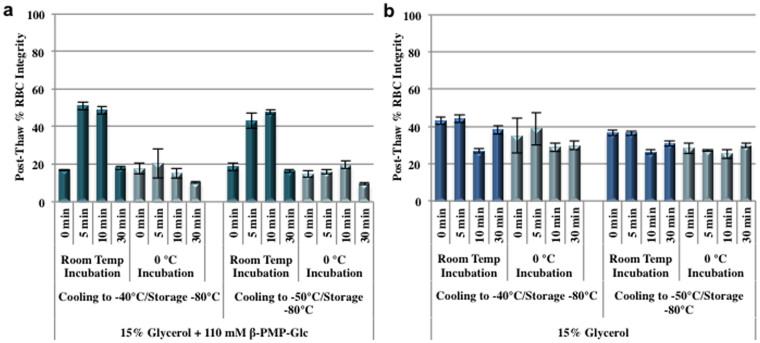
Pre-incubation time with β-PMP-Glc. The effect of pre-incubation time of cryo-solutions on post-thaw RBC integrity. (a) RBC samples were incubated for indicated time and at indicated temperature with a 110 mM β-PMP-Glc + 15% glycerol solution prior to freezing using an interrupted freezing protocol. (b) RBC samples were incubated for indicated time and at indicated temperature with a 15% glycerol solution prior to freezing using an interrupted freezing protocol. Results are presented as mean post-thaw RBC integrity (n = 4–30). Error bars represent standard error of the mean (SEM).

The cryoprotective property of glycerol is largely attributed to its ability to penetrate cells and it is well established that RBCs are highly permeable to glycerol^56^,^57^,^58^. Transport of glucose across human RBC membranes readily occurs through facilitated diffusion, a process that is mediated by glucose transporters (GLUTs) that are expressed in high levels on the surface of RBCs^59^,^60^. However, the uptake of glucose (and glucose derivatives) is dependant upon the structure of the pyranose ring (ie. axial *vs.* equatorial positioning of hydroxyl groups) and ultimately the “shape” of the molecule^61^,^62^. The data from the two-step interrupted freezing experiment presented in the previous section suggests that β-PMP-Glc may be conferring protection against cryo-injury by interacting with RBC membrane and/or penetrating the cell. Consequently, we sought to explore whether the observed increase in post-thaw integrity was the result of internalization of β-PMP-Glc. Towards this end, suspensions of RBCs in 15% glycerol containing 110 mM β-PMP-Glc were incubated for 0, 5, 10 and 30 min time periods at room temperature or 0°C as it has previously been demonstrated that the rate of sugar transport can be considerably slowed at 0°C^63^. After incubation, the samples were frozen by slowly cooling to −40 or −50°C followed by rapid cooling to −80°C prior to thawing. Post-thaw RBC integrities from these experiments are shown in Fig. 4.

When 15% glycerol solutions were used alone (no β-PMP-Glc present) post-thaw RBC integrity typically ranged from 30–40% and was independent of incubation time or cooling temperature (Fig. 4b). In contrast, the optimal incubation times of RBCs with the 15% glycerol/β-PMP-Glc solution is 5 to 10 min at room temperature (Fig. 4a). Post-thaw integrity for these time periods was approximately 50% and was independent of the temperature the sample was cooled to prior to being placed in place in dry ice. Interestingly, a suspension of RBCs with the 15% glycerol/β-PMP-Glc solution followed by immediate freezing (no incubation) yielded RBC integrities less than 20%. The fact that post-thaw integrity is better with a 5 or 10 min incubation suggests that β-PMP-Glc may be internalized. A decrease in post-thaw RBC integrity was observed with longer incubation times (30 min) suggesting a toxic effect with β-PMP-Glc was occurring. However, less than 1% hemolysis (>99% RBC integrity) was observed upon incubation of RBCs with β-PMP-Glc (with or without glycerol) for 60 min at room temperature without freezing (supplementary information, Fig. S2), indicating that hemolysis is not occurring during the incubation stage of the protocol but rather during freezing and/or thawing.

Incubation of RBCs at 0°C in the 15% glycerol/β-PMP-Glc solution for 0, 5 and 10 min prior to freezing also resulted in decreased post-thaw integrity to approximately 20% (Fig. 4a). This drastic decrease at 0°C was only observed when β-PMP-Glc was added to the freezing solution. While the permeability of glycerol into cells is temperature dependent^46^, the rate at which glycerolization occurs in RBCs is fast even at 0°C^57^. Thus, at the lower temperatures and shorter incubation times, the RBCs are still glycerolized prior to freezing. However, the internalization of β-PMP-Glc is likely an ATP-dependent process^59^,^60^ and at 0°C little or no internalization would be expected resulting in poor post-thaw integrity as observed in Fig. 4a. Overall, this data is consistent with the hypothesis that the protective effect of β-PMP-Glc with RBCs is the result of internalization, however further studies are required to determine the quantity and rate of its transport into RBCs.

### Cryomicroscopy with β-PMP-Glc

**Figure 5:**
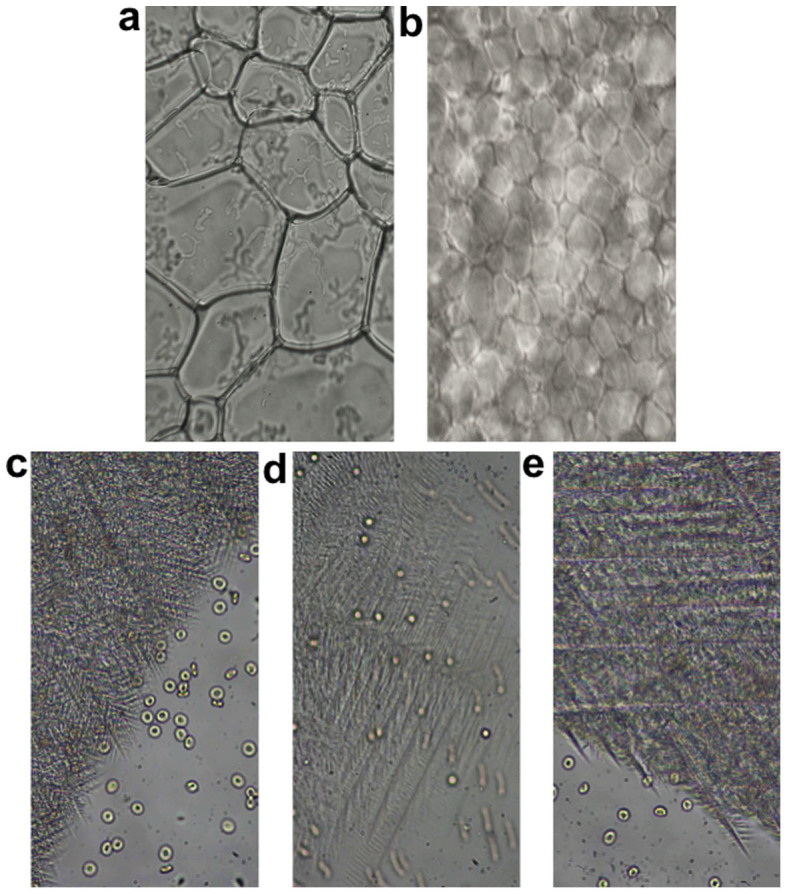
Ice crystal and cryomicroscopy images. Photographs illustrating the IRI activity of β-PMP-Glc (**6**) and the advancing ice-front during RBC freezing. (a–b) Ice crystal grains from a frozen (a) PBS solution, or (b) 22 mM β-PMP-Glc solution in PBS annealed at −6.4°C for 30 mins in a splat-cooling assay. (c–e) Advancing ice fronts during RBC freezing in the presence of (c) 110 mM β-PMP-Glc + 15% glycerol solution, (d) 110 mM β-PMP-Glc in dextrose/saline solution, or (e) 15% glycerol solution.

As β-PMP-Glc is a potent inhibitor of ice recrystallization, it is expected to affect the structure of ice in a frozen sample, ultimately decreasing the overall size of individual ice crystals in a frozen sample. This is anticipated to result in less mechanical damage to the cells and higher post-thaw integrity. Fig. 5a shows images of the ice crystals observed for the phosphate buffered saline (PBS) control used in the IRI assay. PBS is a positive control for ice recrystallization and the ice crystals have an average size of 20–40 *μ*m. In contrast, Fig. 5b depicts ice crystals of a 22 mM β-PMP-Glc (**6**) solution in PBS, the concentration at which IRI activity was assessed (Fig. 1c). The magnification of Figs 5a and 5b are identical and as expected, the average size of ice crystals in the presence of β-PMP-Glc is much smaller.

Cryomicroscopy experiments have also confirmed that the structure of ice during the freezing process with RBCs is also dramatically different when β-PMP-Glc is present. In fact, depending on the cryoprotective solution used, the structure of ice in the frozen sample differs dramatically. Figs 5c–e depict images of RBCs frozen on a Linkam cryostage and imaged using a Nikon 80i microscope. RBCs were frozen in the presence of a 110 mM β-PMP-Glc solution with (5c) and without 15% glycerol (5d). The image in Fig. 5e is of RBCs frozen using only a 15% glycerol solution. These images show the advancing ice-front (photographed during the freezing of the sample) in the different cryoprotective solutions. All three solutions resulted in a very different structure of ice during freezing. The most interesting images are those shown in Fig. 5c and 5e, which are the 15% glycerol solution both with and without β-PMP-Glc, where the ice structure in the presence of β-PMP-Glc is very different and we obtain significantly increased post-thaw RBC integrities. This is the first example where a novel small molecule ice recrystallization inhibitor is confirmed to influence ice structure in the presence of RBCs cells *in vitro*.

### Structure-function studies of other para-substituted phenolic-glucosides

**Figure 6:**
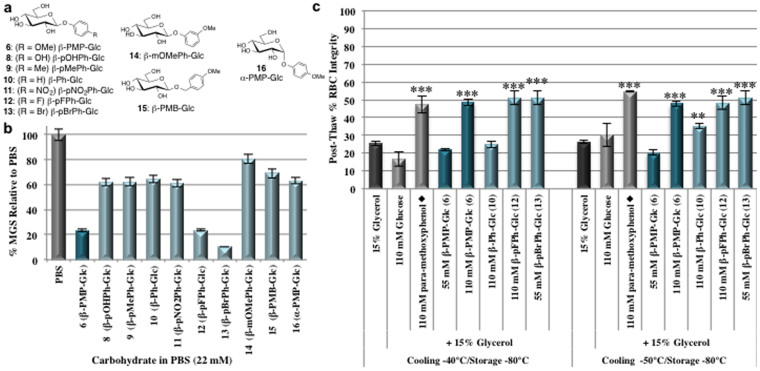
IRI activity and RBC freezing with phenolic-glucosides. (a) Structures of phenolic-glycosides **6** and **8**–**16**. (b) IRI activity of phenolic-glycosides **6** and **8**–**16** at 22 mM in PBS. All compounds are represented as a % MGS (mean grain size) of ice crystals (n = 108) relative to the PBS positive control. (c) RBC freezing using phenolic-glycoside in 15% glycerol. RBC samples were slowly cooled 1°C/min to defined sub-zero temperatures and then rapidly cooled and stored at −80°C. Samples were thawed at 37°C and post-thaw RBC hemolysis was determined by Drabkin's assay. Results are presented as mean (n = 4–30) post-thaw RBC integrity (100 - % hemolysis). Error bars represent standard error of the mean (SEM). ♦ indicates post-thaw Hgb was oxidized to 98% metHgb. Asterisks indicate statistical significance relative to the glycerol control as determined by two-tailed unpaired student's T-test: *, *p* < 0.05; **, *p* < 0.01; ***, *p* < 0.001.

Given that β-PMP-Glc (**6**) conferred protection against cryoinjury in RBCs, we sought to investigate whether other *para*-substituted phenolic glycosides would be effective inhibitors of ice recrystallization and perhaps protect RBCs against injury during freezing. Towards this end, β-linked phenolic-glucoside analogues of **6** were prepared where the aglycone was modified (derivatives **8**–**15**, structures shown in Fig. 6a) or where the configuration of the anomeric carbon was changed to the α-linkage (α-PMP-Glc **16**, Fig. 6a). The preparation of these derivatives is described in the supplementary information and their IRI activity is presented in Fig. 6b. Some of these compounds were also assessed for their potential as cryo-additives for the freezing of RBCs (Fig. 6c). In general, any modifications to β-PMP-Glc (**6**) resulted in reduced IRI activity. For instance, replacing the *para*-methoxy substituent with a hydroxyl, methyl, hydrogen, or nitro group (**8**–**11**, respectively) decreased IRI activity to 60–65% MGS relative to the PBS control from 23% MGS for β-PMP-Glc (Fig. 6b). Similarly, the derivative with a *meta*-substituted methoxy group on the aryl ring (β-mOMePh-Glc, **14**) also exhibited a reduction in IRI activity. The β-linked *para*-methoxybenzyl derivative (β-PMB-Glc, **15**) possessing a methylene spacer between the *O*-glycosidic bond and the aryl ring exhibited a similar decrease in activity. In addition, altering the configuration of the anomeric carbon such that the *para*-methoxyphenyl moiety was attached to glucopyranose residue through an α-linkage (α-PMP-Glc, **16**) rather than a β-linkage also resulted in a decrease in activity (Fig. 6b).

As described earlier in this article, altering the configuration of the C2 or C4 carbons on the glucopyranose decreased IRI as both β-PMP-Gal (**5**) and β-PMP-Man (**7**) were inactive (Fig. 1c). Furthermore, the results presented in Fig. 6b indicate that a β-linkage at the anomeric carbon of the pyranose ring, an aryl ring with direct connection to the C1 oxygen, as well as a methoxy group at the *para*-position on the aryl ring are essential for potent activity. Interestingly, the only modifications that either retain or improve the level of IRI activity associated with β-PMP-Glc (**6**) are ones in which the *para*-methoxy substituent is replaced with a halogen such as fluorine or bromine (Fig. 6b). Derivative **12** (β-pFPhGlc) containing a *para*-fluoro substituent exhibited identical IRI activity to β-PMP-Glc (**6**) with a MGS 23% of the PBS control. This activity was increased to 10% MGS relative to the PBS control with a *para*-bromo substituent (β-pBrPh-Glc, **13**). From these data, a trend between IRI activity and the nature of the *para*-substituent is not apparent. For instance, phenolic-glucosides with substituents that are electron donating were highly IRI active (β-PMP-Glc, **6**) while others were only weakly IRI active (β-pOHPh- and β-pMePh-Glc, **8**–**9**). Similarly, some phenolic glycosides with electron withdrawing substituents exhibited potent activity (β-pFPh- and β-pBrPh-Glc, **12**–**13**) while others had very little IRI activity (β-pNO_2_Ph-Glc, **11**). Further studies are required to probe the relationship between the aromatic substituent and IRI activity with these phenolic-glycosides and the results of these studies will be reported in due course.

Next we sought to probe whether structural modifications that increase the IRI activity of β-PMP-Glc (**6**) also resulted in increased levels of protection during freezing with RBCs. Towards this end, some of the β-linked phenolic glycosides were also utilized as cryo-additives in combination with 15% glycerol. RBCs containing 15% glycerol and these compounds were subjected to the interrupted freezing protocol as described previously^34^,^45^,^46^. In addition, d-glucose and *para*-methoxyphenol, were also examined as they constitute the two structural elements in β-PMP-Glc (**6**). The results are presented in Fig. 6c. When 110 mM glucose in 15% glycerol was utilized the post-thaw RBC integrity is significantly decreased and is comparable to a 15% glycerol solution alone (20–30% intact RBCs). However, when 110 mM *para*-methoxyphenol in 15% glycerol is utilized, post-thaw integrity was similar to that obtained with β-PMP-Glc (~50%). However, upon incubation of RBC samples with *para*-methoxyphenol the supernatants were black/brown in colour. Spectrophotometric analysis indicated that 98% of the hemoglobin (Hgb) in the samples frozen with the *para*-methoxyphenol/glycerol solution was oxidized to methemoglobin (metHgb)^64^. This is undesirable as methemoglobin is unable to bind and transport oxygen^2^.

Replacing the *para*-methoxy substituent of β-PMP-Glc (**6**) with fluorine (β-pFPh-Glc, **12**) or bromine (β-pBrPh-Glc, **13**) increased IRI activity (Fig. 6b). When either of these derivatives was utilized as cryo-additives with 15% glycerol, post-thaw RBC integrities were similar to those with as observed with 110 mM β-PMP-Glc/15% glycerol solution (Fig. 6c). Incubation of RBCs with **12** or **13** in 15% glycerol for 60 min at room temperature without freezing also resulted in less than 1% hemolysis (>99% integrity) indicating that similar to **6**, hemolysis is not occurring during the pre-incubation stage of the freezing protocol (supplementary information, Fig. S2). In contrast, adding the moderately IRI active phenolic-glucoside β-Ph-Glc (**10**, Figs 6a–b) to a 15% glycerol resulted post-thaw RBC integrities that were equivalent to 15% glycerol alone (Fig. 6c). The most surprising result from these studies was observed with the β-pBrPh-Glc derivative (**13**). β-pBrPh-Glc (**13**) was insoluble and precipitated from the cryo-solution when it was utilized at a final concentration of 110 mM in 15% glycerol. Consequently, it was used at 55 mM in 15% glycerol resulting in 50% post-thaw RBC integrity, a result similar to that obtained with 110 mM β-PMP-Glc, but at half the concentration. When 55 mM β-PMP-Glc (**6**) in 15% glycerol was employed as an additive post-thaw integrities were lower than that of the 15% glycerol control (Fig. 6c). Thus, simply replacing the *para*-substituent on the phenolic-glucoside with a bromine atom significantly increased IRI activity (10% MGS for β-pBrPh-Glc **13** compared with 23% for β-PMP-Glc **6**), and resulted in a more effective cryo-additive at a lower concentration.

### β-pBrPh-Glc as a cryo-additive for RBC freezing

**Figure 7:**
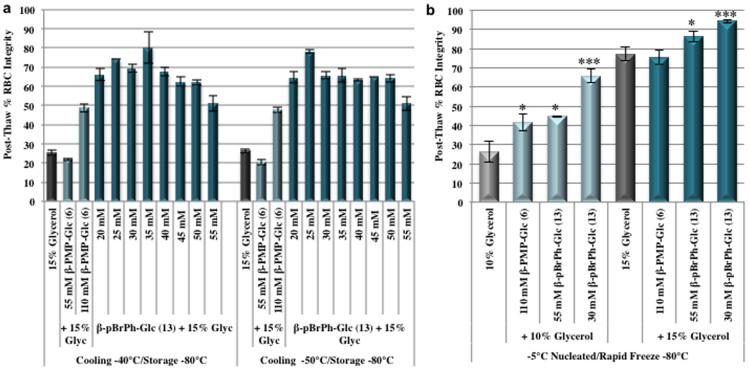
RBC Freezing with reduced β-pBrPh-Glc (13) concentrations. (a) RBC freezing using the interrupted freezing protocol with various concentrations of β-pBrPh-Glc (**13**) containing cryo-solutions in 15% glycerol. RBC samples (n = 4–30) were slow cooled 1°C/min to defined sub-zero temperatures and then rapidly cooled and stored at −80°C. (b) RBC freezing using a rapid-freezing protocol. Samples in indicated cryo-solutions (n = 3–12) were nucleated at -5°C, then rapidly frozen to -80°C by plunging is solid CO_2_ (dry ice). All samples were thawed at 37°C and post-thaw RBC hemolysis was determined by Drabkin's assay. Results are presented as mean post-thaw RBC integrity (100 - % hemolysis). Error bars represent standard error of the mean (SEM). Asterisks indicate statistical significance relative to the glycerol control as determined by two-tailed unpaired student's T-test: *, *p* < 0.05; **, *p* < 0.01; ***, *p* < 0.001.

With the promising results obtained using only 55 mM β-pBrPh-Glc (**13**) in 15% glycerol, we determined the optimal concentration for this cryo-additive (Fig. 7a). By decreasing the concentration of phenolic-glycoside **13** below 55 mM in 15% glycerol, post-thaw RBC integrities could be further increased to 60–80%. This is approximately a three-fold increase in post-thaw RBC integrity compared to the 15% glycerol solution. The optimal concentration of β-pBrPh-Glc (**13**) in a 15% glycerol solution was found to be 25–35 mM, resulting in 70–80% RBC integrity post-thaw (Fig. 7a). These results are significantly higher than the post-thaw integrities obtained with both 55 mM β-pBrPh-Glc in 15% glycerol and 110 mM β-PMP-Glc (**6**) in 15% glycerol (~50% post-thaw RBC integrity) using slow freezing rates.

Recently, it was reported that low molecular weight polymers of polyvinyl alcohol (PVA, 9 kDa) resulted in 40 or 60% post-thaw RBC integrity when used alone or when supplemented in a 215 mg/mL hydroxyethyl starch (HES) solution^33^. The freezing rates were very fast and this is consistent with what is required for non-penetrating cryoprotectants such as HES^14^,^54^,^65^,^66^,^67^. The ability of PVA to reduce post-thaw hemolysis was attributed to its ability to inhibit ice recrystallization. Consequently, we explored whether the novel small molecule ice recrystallization inhibitors that were effective cryo-additives using slow freezing rates were also effective during rapid freezing conditions. These results are presented Fig. 7b. Under rapid freezing conditions (plunging sample directly into dry ice), the addition of 110 mM β-PMP-Glc (**6**) or 55 mM β-pBrPh-Glc (**13**) to either 10 or 15% glycerol increased post-thaw RBC integrity relative to the 10 or 15% glycerol control alone. RBC post-thaw integrity in the presence of the phenolic-glycosides and 10% glycerol were ~45% (compared with 25% for 10% glycerol alone). Similar to what was observed with the slow cooling conditions, post-thaw integrities could be increased with the use of 15% glycerol, and were 75–85% when the phenolic-glycosides were added in the cryo-solution. The most surprising results were obtained when 30 mM β-pBrPh-Glc was added to the cryo-solutions under the rapid freezing conditions. Post-thaw integrities were 65% when added to a 10% glycerol solution and 95% when 30 mM β-pBrPh-Glc was added to a 15% glycerol solution (Fig. 7b). This is significantly higher integrity than the glycerol solutions alone. Thus, not only does 30 mM β-pBrPh-Glc (**13**) significantly increase post-thaw RBC integrity under slow-cooling conditions (Fig. 7a), but it is also a highly beneficial cryo-additive under rapid freezing conditions and can decrease post-thaw RBC hemolysis to 5% (95% RBC integrity, Fig. 7b).

## Discussion

Typical clinical cryopreservation protocols for RBCs in Canada and the United States rely on using high amounts of glycerol (~40%) combined with a slow freezing rate (1°C/min) to −80°C^1^,^7^. Following thawing, deglycerolization must be performed prior to transfusion to ensure intracellular glycerol concentrations are less than 1% to prevent intravascular hemolysis upon transfusion^5^,^7^,^8^,^9^. This process requires specialized equipment and is time-consuming. The results described in this article indicate that small molecule potent ice recrystallization inhibitors can be successfully utilized for the freezing of RBCs in the presence of reduced glycerol concentrations. This was demonstrated with β-PMP-Glc (**6**) and β-pBrPh-Glc (**13**) using both rapid freezing rates (Fig. 7b) and slow freezing rates (Figs 2 and 7a). However, the most promising results from this study arise from the results using slow freezing rates (Figs 2 and 7a). The addition of IRI active phenolic-glucosides either doubled or tripled post-thaw RBC integrity relative to the glycerol control, with the most promising results obtained with 30 mM β-pBrPh-Glc (**13**) in the presence of only 15% glycerol where 70–80% post-thaw RBC integrity was obtained. The slow-cooling conditions employed in these studies closely reflect RBC cryopreservation conditions that are typically clinically employed in Canada and the United States for the cryopreservation of RBCs using high concentrations of glycerol (40%)^1^,^7^.

Both β-PMP-Glc (**6**) and β-pBrPh-Glc (**13**) were very effective inhibitors of ice recrystallization (Figs 1c and 6b) and changed the structure of ice in the frozen sample with RBCs (Fig. 5) and this ability likely plays an important role in the cryopreservation potential of this class of compound with RBCs. Other phenolic-glycosides that were incapable of inhibiting ice recrystallization failed to enhance RBC post-thaw integrity (Fig. 6). In addition, results from the interrupted freezing protocol experiments and pre-incubation studies suggest that β-PMP-Glc (**6**) may be penetrating the RBC membrane much like a cell penetrating cryoprotectant and that internalization is beneficial for the cryoprotective effect (Figs 3–4). However, further studies are required to quantify the internalization of the phenolic-glycosides in this study, as well as to fully elucidate the relationship between IRI activity and effective cryo-additives for RBC freezing.

The results from this work are highly significant as reducing glycerol concentrations from the 40% solutions that are currently employed for RBC cryopreservation can drastically reduce deglycerolization times allowing for faster access to cryopreserved RBC units for life-saving transfusions. Optimization of deglycerolization conditions for these novel RBC cryo-solutions containing β-PMP-Glc or β-pBrPh-Glc additives are currently ongoing and will be reported in due course. Finally, these results constitute the first reported example of novel small molecule ice recrystallization inhibitors that are able to increase post-thaw integrities of human RBCs using lower glycerol concentrations and slow freezing rate protocols.

## Methods

All methods were carried out in accordance with approved guidelines.

### Preparation of phenolic-glycosides

Experimental procedures and spectroscopic data for the preparation of phenolic-glycosides **5**–**16** are described in the supplementary methods and Figs. S3–S7 in the supplementary information. Spectroscopic data for phenolic-glycosides **5**–**16** assessed for IRI activity and utilized in RBC freezing experiments are presented in Figs S8–S22 in the supplementary information. Carbohydrate derivatives **1**–**4** were prepared and assessed for IRI activity as described previously by our laboratory^25^.

### Ice Recrystallization Inhibition (IRI) Activity

Sample analysis for IRI activity was performed using the “splat cooling” method as previously described^41^. All carbohydrate derivatives assessed were dissolved in phosphate buffered saline (PBS) solution and a 10 *μ*L droplet of this solution was dropped from a micropipette through a two meter high plastic tube (10 cm in diameter) onto a block of polished aluminum precooled to approximately −80°C. The droplet froze instantly on the polished aluminum block and was approximately 1 cm in diameter and 20 *μ*m thick. This wafer was then carefully removed from the surface of the block and transferred to a cryostage held at −6.4°C for annealing. After a period of 30 min, the wafer was photographed between crossed polarizing filters using a digital camera (Nikon CoolPix 5000) fitted to the microscope. A total three drops for each sample were assayed and three images were taken from each wafer with the area of twelve crystals in each image being quantified (n = 36 crystals per drop; n = 108 crystals per sample). Image analysis of the ice wafers was performed using a novel domain recognition software (DRS) program^42^. This processing employed the Microsoft Windows Graphical User Interface to allow a user to visually demarcate and store the vertices of ice domains in a digital micrograph. The data was then used to calculate the domain areas. All data was plotted and analyzed using Microsoft Excel. The mean grain (or ice crystal) size (MGS) of the sample was compared to the MGS of the control PBS solution for that same day of testing. IRI activity is reported as the percentage of the MGS (% MGS) relative to the PBS control, and the % MGS for each sample was plotted along with its standard error of the mean. Small percentages represent a small MGS (small ice crystals), which is indicative of high IRI activity.

### Blood Collection and Preparation

All RBC units were obtained from NetCAD (Canadian Blood Services' Network Centre for Applied Development). Whole blood was collected from healthy volunteers using standardized phlebotomy guidelines approved by Canadian Blood Services. Informed consent was obtained from all donors. All experimental protocols were approved by NetCAD and Canadian Blood Services (CBS). Ethics approvals were obtained from Research Ethics Board (REB) at CBS and the University of Alberta. Whole blood was centrifuged at 2200 g for 10 min at 4°C. Plasma supernatant and buffy coat were removed and the RBC pellet was washed twice with 0.2%/0.9% dextrose/saline buffer. After the last wash, RBCs were resuspended in an equal volume of packed RBCs of 0.2%/0.9% dextrose/saline buffer to a final hematocrit (Hct) of 0.50 L/L. RBC samples in buffer solution were stored refrigerated (4–6°C) for up to 1 week until needed.

### RBC Freezing Experiments

An equal volume of freezing solution in 0.2%/0.9% dextrose/saline buffer was added to 150 *μ*L of leukoreduced RBCs in 0.2%/0.9% dextrose/saline buffer for a final volume of 300 *μ*L. The final concentrations of all freezing solutions were as indicated in the results and discussion. RBC suspensions were transferred to cryo-tubes and incubated at room temperature for 10 min prior to immersion in a methanol bath cooled to −5°C. A thermocouple was inserted into a glycerol control sample for temperature measurements at 1 s intervals. Once the internal solution reached −5°C, ice nucleation was induced by touching the cryo-tubes with pre-cooled (in liquid nitrogen) forceps. RBC samples were then held at −5°C for 5 min. For slow freezing conditions, samples were then cooled at a rate of 1°C/min to defined subzero temperatures (see text), then either thawed immediately from this temperature (“cooling/immediate thawing” profiles) or plunged into dry ice (−80°C) or liquid nitrogen (−196°C) for 30 min (“cooling/storage” profiles) and stored. For rapid freezing conditions, following nucleation samples were plunged into dry ice (−80°C) and stored for a minimum of 30 minutes prior to thawing.

### Thawing and Post-Thaw Hemolysis

All RBC samples were thawed under fast-thaw conditions in a 37°C water bath. Post-thaw Hcts and percent hemolysis was determined for all freezing experiments by comparing the supernatant hemoglobin concentration to total hemoglobin concentration using the cyanmethemoglobin Drabkin's method^47^,^48^. Percent post-thaw RBC integrity was calculated using the measured percent hemolysis values according to the following equation: % post-thaw RBC integrity = 100 - % hemolysis. Data is represented as the mean post-thaw RBC integrity for each condition. Slow freezing conditions to −196°C were repeated two times for each freezing solution (n = 2). Slow freezing conditions to −80°C were repeated four to thirty times (n = 4–30). Rapid freezing conditions were repeated three to twelve times (n = 3–12).

### Data Analysis

All calculations for percent hemolysis, percent RBC integrity, average % MGS were determined using Microsoft Excel. All error bars are reported as the standard error of the mean (SEM). Statistical significance for all data was determined by unpaired Student's t-test with a 95%, 99% or 99.9% confidence level.

### Cryomicroscopy

Cryomicroscopy was performed using a Linkam FDCS cryostage and temperature controllers that were fitted to a Nikon 80i microscope. A small volume (10 *μ*L) of sample was added to a quartz crucible, placed on the cryostage and cooled at 25°C/min to a pre-determined temperature that was 5°C below the estimated freezing point for the sample. Ice was nucleated using a liquid nitrogen-cooled probe and the ice front was collected in real time using a Hamamatsu Photonics ORCA II camera and Nikon NIS-Elements software.

## Author Contributions

C.J.C. and R.S.M. conducted the chemical synthesis and IRI analysis. C.J.C., J.D.R.K. and T.R.T. conducted the RBC freezing experiments. R.N.B. and J.P.A. conceived the project. R.N.B., C.J.C., J.D.R.K., T.R.T. and J.P.A. designed the RBC experiments and interpreted the data. C.J.C. and R.N.B. wrote the paper with input from J.D.R.K., T.R.T. and J.P.A.

## Supplementary Material

Supplementary Information Supplementary Information
