# Supplementary material for: Small Molecule Ice Recrystallization Inhibitors Enable Freezing of Human Red Blood Cells with Reduced Glycerol Concentrations
Source: Sci Rep. 2015 Apr 8;5:9692. doi: 10.1038/srep09692 (PMC4389209; doi:10.1038/srep09692)
Supplement: Supplementary Information [file srep09692-s1.pdf]

Supplementary Information

Small Molecule Ice Recrystallization Inhibitors Enable Freezing of Human Red Blood Cells with Reduced Glycerol Concentrations

Chantelle J. Capicciotti[1], Jayme D. R. Kurach[2], Tracey R. Turner[2], Ross S. Mancini[1], Jason P. Acker[2] and Robert N. Ben[1,*]

[1]*Department of Chemistry, D'Iorio Hall, 10 Marie Curie, University of Ottawa, Ottawa, ON, Canada, K1N 6N5 Fax: 1-613-562-5170 Tel:1-613-562-5800; E-mail: rben@uottawa.ca.*

[2]*Canadian Blood Services, Centre for Innovation, 8249 – 114 Street NW, Edmonton, AB, Canada, T6G 2R8 Tel: 1-780-702-8269; E-mail: jason.acker@blood.ca.*

<u>Table of Contents</u>

Supplemental RBC Freezing Data 2

Supplementary Methods 3

General Experimental: 3

Synthesis of p-methoxyphenyl-β-D-galactopyranoside (5) and p-methoxyphenyl-β-D-glucopyranoside (6): 4

Synthesis of p-methoxyphenyl-β-D-mannopyranoside (7): 5

Synthesis of phenolic-glucopyranosides 8-13: 8

Synthesis of p-methoxybenzyl-glucopyranoside 15: 13

Synthesis of α–linked p-methoxyphenyl-glucopyranoside 16: 14

References 15

Spectra 16

Supplemental RBC Freezing Data

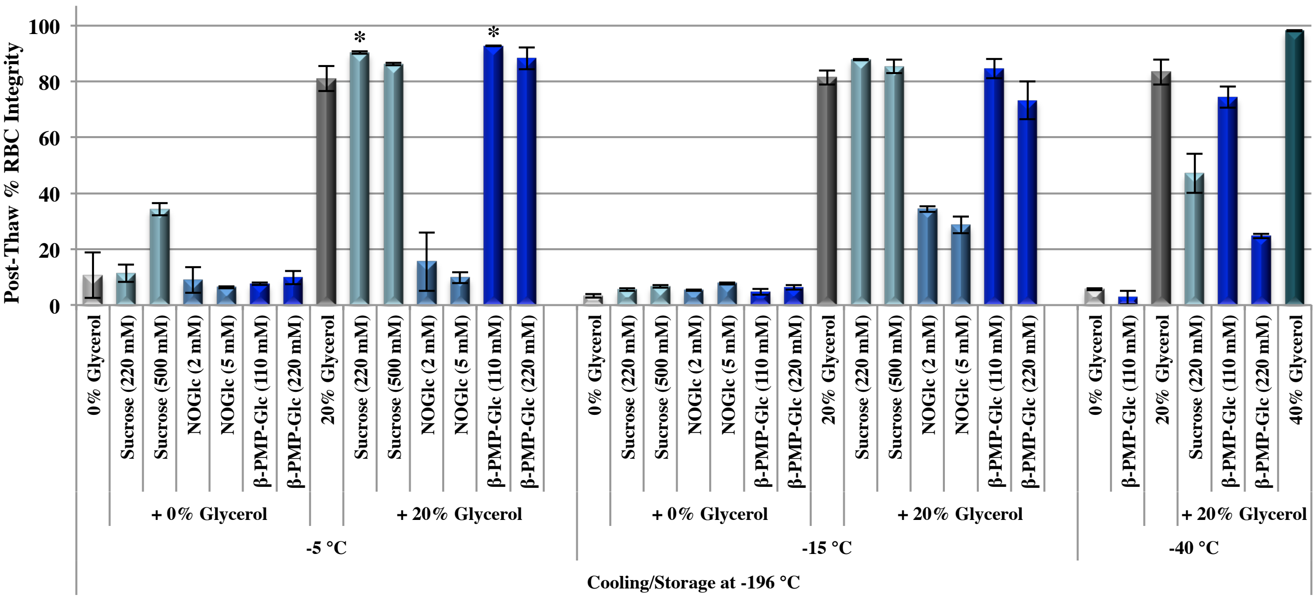

Figure S1 | RBC freezing with novel small molecule IRIs. The effect of NOGlc (**4**), β-PMP-Glc (**6**) and sucrose on post-thaw RBC integrities. RBC samples in indicated cryo-solutions containing no (0%) or 20% glycerol in a dextrose/saline buffer were slow-cooled 1 °C/min to defined sub-zero temperatures and then rapidly cooled (cooling rate approximately 115 ºC/min) by immersion into liquid nitrogen and stored at -196 °C. All samples were quickly thawed at 37 °C and post-thaw hemolysis was quantified post-thaw using Drabkin's assay to determine % RBC integrity (100 - % hemolysis). Error bars represent SEM. Asterisks indicate statistical significance relative to the glycerol control as determined by two-tailed unpaired student's T-test: *, p <0.05; **, p < 0.01; ***, p < 0.001.

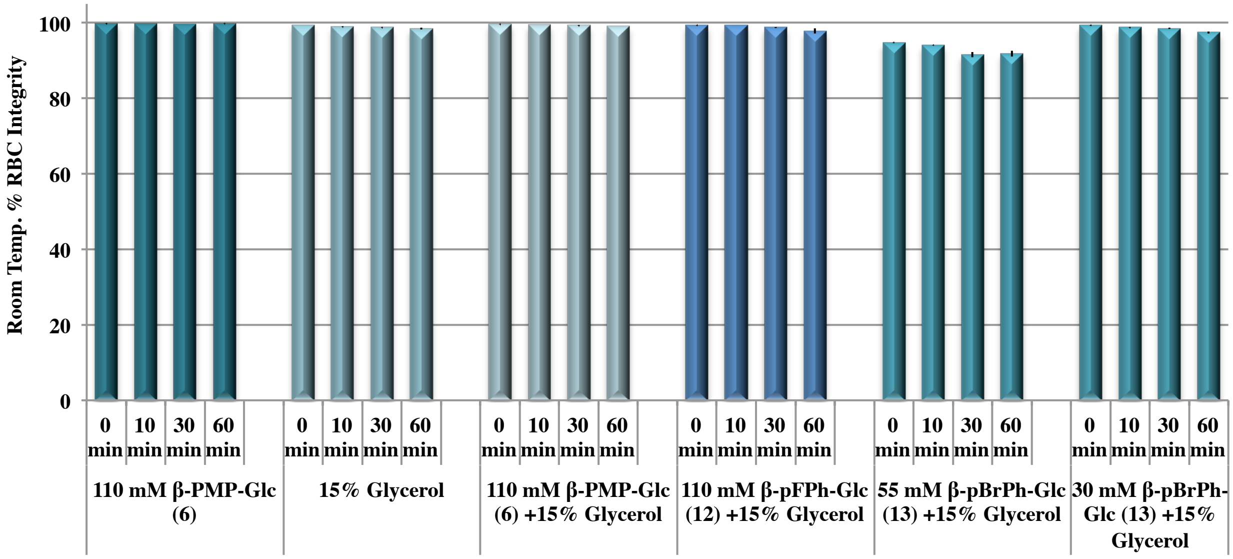

Figure S2 | Toxicity of β-PMP-Glc (6), β-pFPh-Glc (12) and β-pBrPh-Glc (13) cryo-solutions on RBCs. RBC integrities after room temperature incubation for indicated time period with cryo-solutions without freezing. Hemolysis was quantified post-thaw using Drabkin's assay to determine % RBC integrity (100 - % hemolysis). Error bars represent SEM.

Supplementary Methods

General Experimental:

All anhydrous reactions were performed in flame-dried glassware under a positive pressure of dry argon. Air or moisture-sensitive reagents and anhydrous solvents were transferred with oven-dried syringes or cannulae. All flash chromatography was performed with E. Merck silica gel 60 (230-400 mesh). All solution phase reactions were monitored using analytical thin layer chromatography (TLC) with 0.2 mm pre-coated silica gel aluminum plates 60 F254 (E. Merck). Components were visualized by illumination with a short-wavelength (254 nm) ultra-violet light and/or staining (ceric ammonium molybdate, potassium permanganate, or phosphomolybdate stain solution). All solvents used for anhydrous reactions were distilled. Tetrahydrofuran (THF) and diethyl ether (Et_2O) were distilled from sodium/benzophenone under nitrogen. Dichloromethane (DCM) was distilled from calcium hydride. *N,N*-dimethylformamide (DMF) was stored over activated 4Å molecular sieves under argon. ^{1}H (400 or 500 MHz) and ^{13}C NMR (100 or 125 MHz) spectra were recorded at ambient temperature on a Bruker Avance 400, Bruker Avance 500, or Varian Inova 500 spectrometer. Deuterated chloroform ($CDCl_3$) or water (D_2O) were used as NMR solvents, unless otherwise stated. Chemical shifts are reported in ppm using the solvent residual peak as an internal standard. Splitting patterns are designated as follows: s, singlet; d, doublet; t, triplet; q, quartet; quint, quintet; m, multiplet and br, broad. Low resolution mass spectrometry (LRMS) was performed on a Micromass Quatro-LC Electrospray spectrometer with a pump rate of 20 μL/min using electrospray ionization (ESI).

Compounds below are in order of appearance in the manuscript. Intermediates that were not numbered in the manuscript received numbers beginning with **12**. NMR spectra for novel compounds and final compounds assessed for IRI activity are provided.

Synthesis of p-methoxyphenyl-β-D-galactopyranoside (5) and p-methoxyphenyl-β-D-glucopyranoside (6):

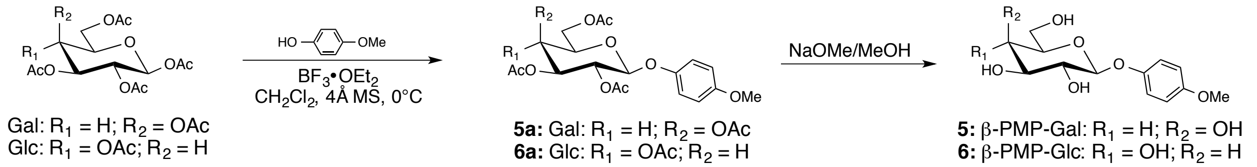

Figure S3. Synthesis of β-PMP-Gal (**5**) and β-PMP-Glc (**6**)

p-Methoxyphenyl-2,3,4,6-tetra-*O*-acetyl-β-D-galactopyranoside (5a)

To a mixture of 1,2,3,4,6-penta-*O*-acetyl-β-D-galactopyranose (5.0 g, 12.8 mmol), 4-methoxyphenol (2.22 g, 17.9 mmol) and 4 Å MS in anhydrous CH_2Cl_2 (50 mL) stirring at 0 °C under Ar, was slowly added boron trifluoride diethyl etherate (2.09 mL, 16.6 mmol). The reaction mixture was stirred overnight, then diluted with CH_2Cl_2 and quenched with sodium bicarbonate. The solution was filtered through Celite®, then extracted with CH_2Cl_2. The organic layer was washed with sodium bicarbonate, water, saturated brine, then dried over $MgSO_4$ and concentrated. Column chromatography (3:1 hexanes/ethyl acetate) afforded **5a** as a white powder (5.3 g, 91%). Characterization data is consistent with that previously reported[1]. ^{1}H NMR (500 MHz, $CDCl_3$): δ 6.97-6.94 (m, 2H), 6.84-6.80 (m, 2H), 5.47-5.44 (m, 2H), 5.09 (dd, *J* = 10.4, 3.4 Hz, 1H), 4.92 (d, *J* = 8.0 Hz, 1H), 4.23 (dd, *J* = 11.3, 6.8 Hz, 1H), 4.16 (dd, *J* = 11.3, 6.5 Hz, 1H), 4.02-3.99 (m, 1H), 3.78 (s, 3H), 2.18 (s, 3H), 2.09 (s, 3H), 2.06 (s, 3H), 2.01 (s, 3H). ^{13}C NMR (100 MHz, $CDCl_3$): δ 170.3, 170.0, 169.4, 169.3, 155.8, 150.9, 118.7, 114.5, 100.7, 72.4, 71.3, 70.2, 66.3, 61.4, 55.5, 20.7, 20.6, 20.6, 20.5. LRMS (ESI): *m/z* calcd. for $C_{21}H_{30}NO_{11}$ $[M+NH_4]^+$ 472.5; found, 472.3.

p-Methoxyphenyl-β-D-galactopyranoside (5)

Compound **5a** (5.3 g, 11.6 mmol) was dissolved in a solution of sodium methoxide in methanol (25 mL) and stirred for one hour at room temperature. The solution was then neutralized with Amberlite® IR-120 (H^+) ion-exchange resin and filtered. The filtrate was concentrated and the product was lyophilized to yield **5** as a white powder (3.3 g, 98%). Characterization data is consistent with that previously reported[1]. ^{1}H NMR (400 MHz, CD_3OD): δ 7.08-7.04 (m, 2H), 6.85-6.81 (m, 2H), 4.72 (d, *J* = 7.7 Hz, 1H), 3.89 (dd, *J* = 3.4, 0.8 Hz, 1H), 3.78-3.74 (m, 3H), 3.74 (s, 3H), 3.63 (ddd, *J* = 6.7, 5.5, 1.1 Hz, 1H), 3.55 (dd, *J* = 9.7, 3.4 Hz, 1H). ^{13}C NMR (101 MHz, D_2O): δ 154.6, 151.0, 118.1, 115.0, 101.7, 75.4, 72.6, 70.6, 68.5, 60.7, 55.8. LRMS (ESI): *m/z* calcd. for $C_{13}H_{18}NaO_7$ $[M+Na]^+$ 309.3; found, 309.3.

p-Methoxyphenyl-2,3,4,6-tetra-*O*-acetyl-β-D-glucopyranoside (6a)

To a mixture of 1,2,3,4,6-penta-*O*-acetyl-β-D-glucopyranose (15 g, 38.4 mmol), 4-methoxyphenol (6.7 g, 53.8 mmol) and 4 Å MS in anhydrous CH_2Cl_2 (100 mL) stirring at 0 °C under Ar, was slowly added boron trifluoride diethyl etherate (9.64 mL, 76.8 mmol). The reaction mixture was stirred overnight, then diluted with CH_2Cl_2 and

quenched with sodium bicarbonate. The solution was filtered through Celite®, then extracted with CH_2Cl_2. The organic layer was washed with sodium bicarbonate, water, saturated brine, then dried over $MgSO_4$ and concentrated. Column chromatography (6:4 hexanes/ethyl acetate) afforded **6a** as a white powder (13.6 g, 78%). Characterization data is consistent with that previously reported[2]. ^{1}H NMR (300 MHz, $CDCl_3$): δ 6.97-6.92 (m, 2H), 6.84-6.78 (m, 2H), 5.31-5.20 (m, 2H), 5.16 (t, *J* = 9.6 Hz, 1H), 4.95 (d, *J* = 7.6 Hz, 1H), 4.29 (dd, *J* = 12.3, 5.2 Hz, 1H), 4.18 (dd, *J* = 12.3, 2.5 Hz, 1H), 3.83-3.79 (m, 1H), 3.77 (s, 3H), 2.08 (s, 3H), 2.07 (s, 3H), 2.04 (s, 3H) 2.03 (s, 3H). ^{13}C NMR (75 MHz, $CDCl_3$): δ 170.6, 170.3, 169.4, 169.3, 155.8, 150.9, 118.7, 114.5, 100.3, 72.7, 71.9, 71.2, 68.3, 61.9, 55.6, 20.7, 20.7, 20.6, 20.6. LRMS (ESI): *m/z* calcd. for $C_{21}H_{30}NO_{11}$ $[M+NH_4]^+$ 472.5; found, 472.2; *m/z* calcd. for $C_{21}H_{26}NaO_{11}$ $[M+Na]^+$ 477.4; found, 477.1.

p-Methoxyphenyl-β-D-glucopyranoside (6)

Compound **6a** (6.75 g, 14.9 mmol) was dissolved in a solution of sodium methoxide in methanol (25 mL) and stirred for one hour at room temperature. The solution was then neutralized with Amberlite® IR-120 (H^+) ion-exchange resin, filtered and concentrated. The filtrate was concentrated and the product was lyophilized to yield **6** as a white powder (4.1 g, 95%). ^{1}H NMR (400 MHz, D_2O): δ 7.14-7.10 (m, 2H), 7.01-6.96 (m, 2H), 5.01 (d, *J* = 7.6 Hz, 1H), 3.92 (dd, *J* = 12.4, 2.2 Hz, 1H), 3.81 (s, 3H), 3.75 (dd, *J* = 12.4, 5.7 Hz, 1H), 3.62-3.54 (m, 3H), 3.48 (dd, *J* = 9.6, 9.2 Hz, 1H). ^{13}C NMR (101 MHz, D_2O): δ 154.7, 150.9, 118.2, 115.0, 101.2, 76.1, 75.5, 72.9, 69.4, 60.5, 55.8. LRMS (ESI): *m/z* calcd. for $C_{13}H_{18}NaO_7$ $[M+Na]^+$ 309.3; found, 309.3.

Synthesis of p-methoxyphenyl-β-D-mannopyranoside (7):

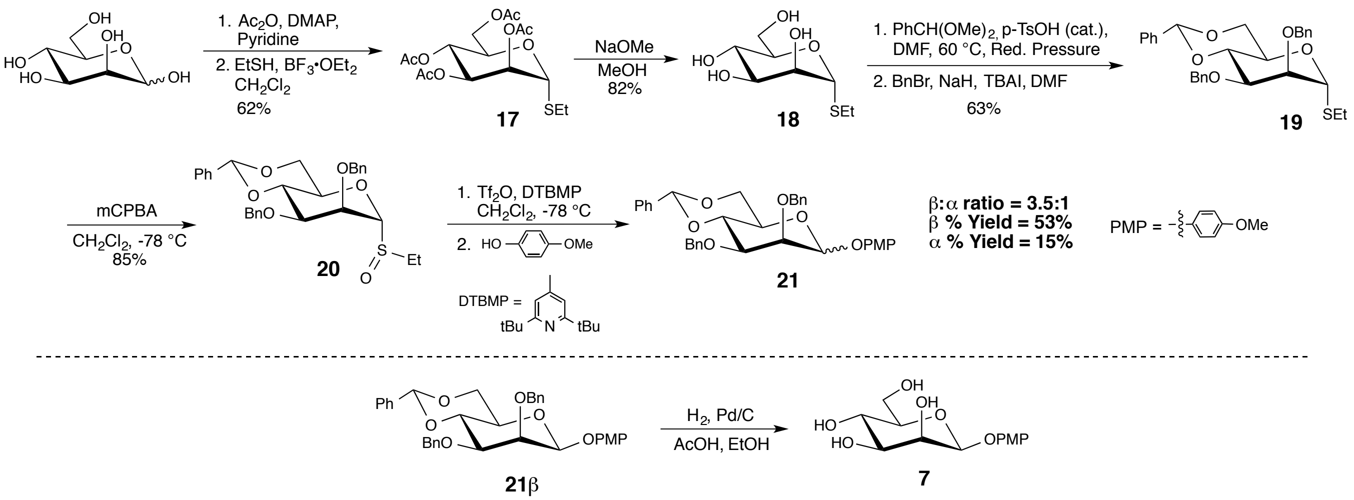

Figure S4. Synthesis of β-PMP-Man (**7**).

Ethyl 2,3,4,6-Tetra-*O*-Acetyl-1-thio-α-D-Mannopyranoside (17)

To a solution of D-mannose (2.0 g, 11.1 mmol) in dry pyridine (45 mL) was added acetic anhydride (26 mL) and the reaction mixture was stirred at 0 °C for 1 hour. A catalytic amount of 4-dimethylaminopyridine was added and the mixture was stirred at room temperature overnight. Ethanol was added and the solvent was evaporated under *in vacuo* and the residue was diluted in ethyl acetate, washed with sodium bicarbonate, water and brine. The mixture was dried with $MgSO_4$, filtered and concentrated. The crude product was then added to a solution of ethanethiol (1.23 mL, 16. 6 mmol) and 4 Å MS in anhydrous CH_2Cl_2 (30 mL) stirring at 0 °C under Ar and boron trifluoride diethyl etherate (4.2 mL, 33.3 mmol) was added dropwise. The reaction mixture was stirred overnight, then diluted with CH_2Cl_2 and quenched with sodium bicarbonate. The solution was filtered through Celite®, then extracted with CH_2Cl_2. The organic layer was washed with sodium bicarbonate, water, saturated brine, then dried over $MgSO_4$ and concentrated. Column chromatography (7:3 hexanes/ethyl acetate) afforded **17** as a syrup (2.7 g, 62%). Characterization data is consistent with that previously reported[3]. ^{1}H NMR (300 MHz, $CDCl_3$): δ 5.34 (dd, *J* = 3.2, 1.6 Hz, 1H), 5.31-5.27 (m, 3H), 4.40 (td, *J* = 7.1, 2.5 Hz, 1H), 4.32 (dd, *J* = 12.1, 5.3 Hz, 1H), 4.10 (dd, *J* = 12.1, 2.2 Hz, 1H), 2.71-2.57 (m, 2H), 2.17 (s, 3H), 2.10 (s, 3H), 2.05 (s, 3H), 1.99 (s, 3H), 1.31 (t, *J* = 7.4 Hz, 3H). ^{13}C NMR (75 MHz, $CDCl_3$): δ 170.6, 170.0, 169.8, 169.7, 82.2, 71.1, 69.5, 68.9, 66.3, 62.4, 25.4, 20.9, 20.7, 20.7, 20.60, 14.7. LRMS (ESI): *m/z* calcd. for $C_{16}H_{24}NaO_9S$ $[M+Na]^+$ 415.4; found, 415.2.

Ethyl 1-thio-α-D-Mannopyranoside (18)

Compound **17** (2.7 g, 6.9 mmol) was dissolved in a solution of sodium methoxide in methanol (10 mL) and stirred for one hour at room temperature. The solution was neutralized with Amberlite® IR-120 (H^+) ion-exchange resin, filtered and concentrated to yield **18** as a solid (1.5 g, 95%). Characterization data is consistent with that previously reported[4]. ^{1}H NMR (300 MHz, $CDCl_3$): δ 5.31 (d, *J* = 1.5 Hz, 1H), 4.03 (dd, *J* = 3.3, 1.6 Hz, 1H), 4.01-3.96 (m, 1H), 3.87 (dd, *J* = 12.3, 2.3 Hz, 1H), 3.79-3.72 (m, 2H), 3.65 (t, *J* = 9.7 Hz, 1H), 2.75-2.57 (m, 2H), 1.26 (t, *J* = 7.4 Hz, 3H). ^{13}C NMR (75 MHz, $CDCl_3$): δ 84.2, 73.0, 71.8, 71.0, 67.1, 60.8, 24.7, 14.0. LRMS (ESI): *m/z* calcd. for $C_8H_{15}O_5S$ $[M-H]^-$ 223.3; found, 223.0.

Ethyl 4,6-*O*-Benzylidene-2,3-*O*-Benzyl-1-thio-α-D-Mannopyranoside (19)

To a solution of **18** (375 mg, 1.67 mmol) in DMF (3 mL) was added benzaldehyde dimethyl acetal (251 μL, 1.67 mmol) and catalytic *p*-toluenesulfonic acid. The solution was stirred under reduced pressure at 60 °C for 3 hours then cooled to room temperature and triethylamine (50 μL) was added. The mixture was then evaporated and crystallized from CH_2Cl_2/pet. ether to afford 360 mg (69%) of the crude product that was used without further purification. The crude product (166 mg, 0.53 mmol) was dissolved in DMF (5 mL) and to this solution was added NaH (85 mg, 2.12 mmol) and the mixture was stirred for 10 min. Benzyl bromide (189 μL, 1.59 mmol) was added, followed by a catalytic amount of tetrabutylammonium iodide and the reaction mixture was stirred

overnight. The mixture was quenched the following day with brine and diluted with ethyl acetate. The organic layer was extracted and washed with brine, then dried over $MgSO_4$ and concentrated. Purification by flash chromatography (19:1 hexanes/ethyl acetate) yielded **19** (221 mg, 91%, 63% overall) as a syrup. Characterization data is consistent with that previously reported in the literature[5]. ^{1}H NMR (300 MHz, $CDCl_3$): δ 7.53-7.50 (m, 2H), 7.39-7.28 (m, 13H), 5.65 (s, 1H), 5.31 (d, *J* = 0.8 Hz, 1H), 4.82-4.71 (m, 3H), 4.63 (d, *J* = 12.2 Hz, 1H), 4.32-4.16 (m, 3H), 3.94-3.87 (m, 3H), 2.68-2.48 (m, 2H), 1.24 (t, *J* = 7.4 Hz, 3H). ^{13}C NMR (76 MHz, $CDCl_3$): δ 138.4, 137.9, 137.6, 128.8, 128.4, 128.3, 128.2, 128.1, 127.8, 127.6, 127.5, 126.1, 101.4, 83.5, 79.2, 78.1, 76.4, 73.1, 73.0, 68.6, 64.6, 25.3, 14.9. LRMS (ESI): *m/z* calcd. for $C_{29}H_{33}O_5S$ $[M+H]^+$ 493.6; found, 493.2.

Ethyl 4,6-*O*-Benzylidene-2,3-*O*-Benzyl-1-thio-α-D-Mannopyranoside S-Oxide (20)

To a stirred solution of **19** (89 mg, 0.181 mmol) in dry dichloromethane (3 mL) at-78 °C was added 70% *meta*-chloroperbenzoic acid (41 mg, 0.181 mmol). The reaction mixture was stirred at -78 °C for 4 hours then warmed to -20 °C and quenched with sodium carbonate. The organic layer was extracted and washed with Na_2CO_3, water and brine, then dried over $MgSO_4$ and concentrated. Purification by column chromatography (2:1 Hex/EtOAc) yielded **20** (78 mg, 85%). Characterization data is consistent with that previously reported in the literature[6]. ^{1}H NMR (300 MHz, $CDCl_3$): δ 7.47-7.25 (m, 15H), 5.61 (s, 1H), 4.85-4.77 (m, 2H), 4.71-4.64 (m, 2H), 4.59 (d, *J* = 1.4 Hz, 1H), 4.49 (dd, *J* = 3.4, 1.4 Hz, 1H), 4.32 (dd, *J* = 10.0, 9.0 Hz, 1H), 4.18 (dd, *J* = 9.8, 4.2 Hz, 1H), 4.10 (dd, *J* = 10.1, 3.4 Hz, 1H), 3.81-3.67 (m, 2H), 2.93-2.83 (m, 1H), 2.68-2.56 (m, 1H), 1.33 (t, *J* = 7.5 Hz, 3H). ^{13}C NMR (76 MHz, $CDCl_3$): δ 138.7, 137.4, 137.2, 128.6, 128.3, 128.3, 128.0, 127.9, 127.8, 127.6, 127.5, 125.6, 101.9, 92.2, 78.2, 77.1, 76.4, 73.1, 73.0, 68.3, 64.6, 45.4, 6.9. LRMS (ESI): *m/z* calcd. for $C_{29}H_{32}KO_6S$ $[M+K]^+$ 547.7; found, 547.1.

***p*-Methoxyphenyl-4,6-*O*-Benzylidene-2,3-*O*-Benzyl-β-D-Mannopyranoside (21)**

Glycosidation conditions to form the β-linked mannopyranoside were as described by Crich et al[7,8]. To a stirred solution of sulfoxide **20** (17.6 mg, 0.035 mmol) and 2,6-di-*tert*-butyl-4-methylpyridine (14.2 mg, 0.069 mmol) in dry dichloromethane (1 mL) cooled to –78 °C under argon was added trifluoromethanesulfonic anhydride (6.4 μL, 0.038 mmol). After stirring for 2-5 minutes a solution of 4-methoxyphenol (4.7 mg, 0.038 mmol) in dry dichloromethane (1 mL) was added dropwise. The reaction mixture was stirred at -78 °C for 2 hours then warmed to 0 °C over 2 hours and maintained at 0 °C for 30 minutes before quenching with sodium bicarbonate, washing with brine, drying over $MgSO_4$ and concentrating. Purification by column chromatography over a gradient (19:1 hexanes/ethyl acetate to 8:2 hexanes/ethyl acetate) gave a 3.5:1 β:α mixture and yielded pure β-anomer (as determined by ^{1}H NMR analysis and $J_{1,2}$ values[9]) (10.1 mg, 53%) and pure α-anomer (2.9 mg, 15%). Characterization data for **21 β-anomer**: ^{1}H-NMR (500 MHz; $CDCl_3$): δ 7.55-7.50 (m, 4H), 7.40-7.28 (m, 11H),

6.95-6.93 (m, 2H), 6.83-6.81 (m, 2H), 5.65 (s, 1H), 5.08 (d, *J* = 12.3 Hz, 1H), 5.01 (d, *J* = 12.2 Hz, 1H), 4.96 (d, *J* = 0.6 Hz, 1H), 4.75 (d, *J* = 12.5 Hz, 1H), 4.65 (d, *J* = 12.4 Hz, 1H), 4.34 (dt, *J* = 10.4, 5.2 Hz, 1H), 4.29 (d, *J* = 9.7 Hz, 1H), 4.11 (d, *J* = 3.0 Hz, 1H), 3.98 (t, *J* = 10.3 Hz, 1H), 3.77 (s, 3H), 3.68 (dd, *J* = 9.9, 3.1 Hz, 1H), 3.46-3.41 (m, 1H). ^{13}C NMR (126 MHz; $CDCl_3$): δ 155.3, 151.0, 138.2, 138.2, 137.5, 128.9, 128.7, 128.3, 128.2, 128.2, 127.7, 127.6, 127.6, 126.0, 117.9, 114.5, 101.5, 100.8, 78.5, 77.8, 76.0, 75.0, 72.6, 68.6, 67.6, 55.6. LRMS (ESI): *m/z* calcd. for $C_{34}H_{34}KO_7$ $[M+K]^+$ 593.7; found, 593.3.

_p_-Methoxyphenyl-β-D-Mannopyranoside (7)

A solution of **21β** (8.6 mg, 0.016 mmol) in 4 mL of EtOH/AcOH (3:1) and 5% Pd/C was stirred for 16 hours under an atmosphere of H_2. The flask was purged with air, the catalyst was removed by filtration through Celite® and the solvents were removed *in vacuo*. Purification by C-18 solid-phase extraction cartridge over a gradient (water to 9:1 water/ACN to 8:2 water/ACN to 1:1 water/ACN) afforded **7** (3.3 mg, 74%) as a white solid. ^{1}H NMR (500 MHz, D_2O): δ 7.11 (d, *J* = 9.1 Hz, 2H), 7.00 (d, *J* = 9.1 Hz, 2H), 5.28 (s, 1H), 4.20 (d, *J* = 2.9 Hz, 1H), 3.94 (dd, *J* = 12.3, 2.2 Hz, 1H), 3.82 (s, 3H), 3.79-3.74 (m, 2H), 3.68 (t, *J* = 9.7 Hz, 1H), 3.51 (ddd, *J* = 9.3, 6.5, 2.5 Hz, 1H). ^{13}C NMR (126 MHz, D_2O): δ 154.4, 150.5, 117.7, 114.9, 98.3, 76.2, 72.7, 70.5, 66.5, 60.8, 55.7. LRMS (ESI): *m/z* calcd. for $C_{13}H_{17}O_7$ $[M-H]^-$ 285.3; found, 285.1.

Synthesis of phenolic-glucopyranosides 8-13:

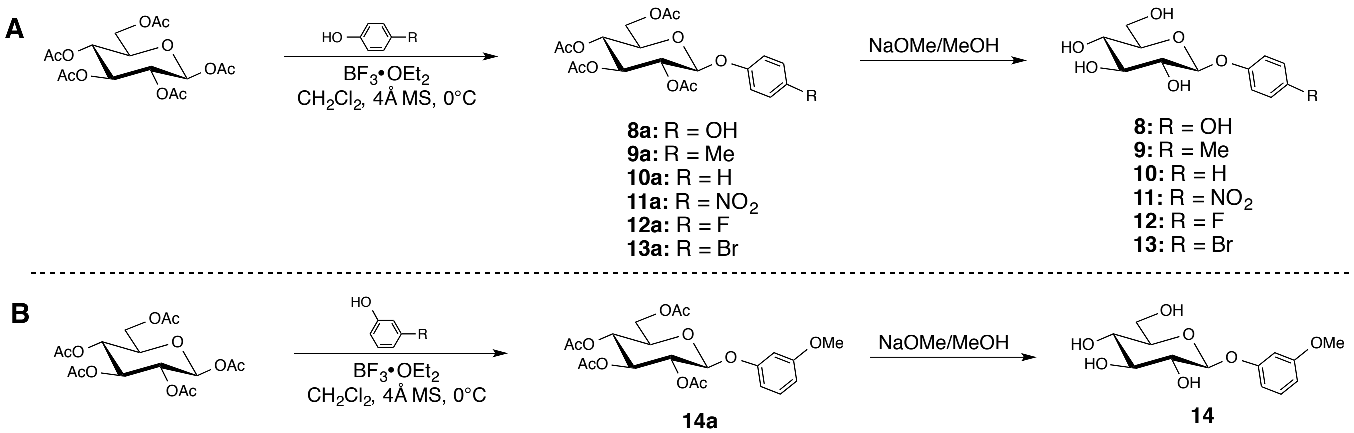

Figure S5. Synthesis of phenolic-glucopyranosides (**A**) **7**-**13** and (**B**) **14**.

_p_-Hydroxyphenyl-2,3,4,6-tetra-_O_-acetyl-β-D-glucopyranoside (8a)

Compound **8a** was prepared in a similar manner as **6a** from 1,2,3,4,6-penta-*O*-acetyl-β-D-glucopyranose (1 g, 2.56 mmol), hydroquinone (211 mg, 1.92 mmol) and boron trifluoride diethyl etherate (482 μL, 3.84 mmol) with 4 Å MS in anhydrous CH_2Cl_2 (15 mL) at 0 °C. Column chromatography (7:3 hexanes/ethyl acetate) afforded **8a** (339

mg, 40%) as a white solid. Characterization data is consistent with that previously reported in the literature[10]. ^{1}H NMR (400 MHz, $CDCl_3$): δ 6.90-6.86 (m, 2H), 6.77-6.72 (m, 2H), 5.30-5.20 (m, 2H), 5.16 (t, *J* = 9.5 Hz, 2H), 4.93 (d, *J* = 7.6 Hz, 1H), 4.28 (dd, *J* = 12.3, 5.1 Hz, 1H), 4.16 (dd, *J* = 12.3, 2.5 Hz, 1H), 3.79 (ddd, *J* = 9.9, 5.1, 2.5 Hz, 1H), 2.09 (d, *J* = 1.5 Hz, 3H), 2.07-2.05 (m, 5H), 2.02 (s, 3H). ^{13}C NMR (101 MHz, $CDCl_3$): δ 170.7, 170.3, 169.5, 169.4, 151.8, 150.8, 118.9, 116.0, 100.3, 72.7, 71.9, 71.2, 68.3, 61.9, 20.7, 20.7, 20.6, 20.6. LRMS (ESI): *m/z* calcd. for $C_{20}H_{24}NaO_{11}$ $[M+Na]^+$ 463.4; found, 463.2.

***p*-Hydroxyphenyl-β-D-glucopyranoside (8)**

Compound **8a** (200 mg, 0.45 mmol) was dissolved in a solution of sodium methoxide in methanol (5 mL) and stirred for one hour at room temperature. The solution was then neutralized with Amberlite® IR-120 (H^+) ion-exchange resin, filtered and concentrated. The filtrate was concentrated and the product was lyophilized to yield **8** as a white powder (122 mg, 98%). ^{1}H NMR (400 MHz, D_2O): δ 7.07-7.03 (m, 2H), 6.89-6.85 (m, 2H), 4.99 (d, *J* = 7.6 Hz, 1H), 3.92 (dd, *J* = 12.4, 2.2 Hz, 1H), 3.75 (dd, *J* = 12.5, 5.6 Hz, 1H), 3.61-3.52 (m, 3H), 3.48 (dd, *J* = 9.7, 8.9 Hz, 1H). ^{13}C NMR (101 MHz, D_2O): δ 151.2, 150.4, 118.4, 116.2, 101.3, 76.0, 75.5, 73.0, 69.4, 60.5. LRMS (ESI): *m/z* calcd. for $C_{12}H_{16}NaO_7$ $[M+Na]^+$ 295.3; found, 295.2.

***p*-Methylphenyl-2,3,4,6-tetra-*O*-acetyl-β-D-glucopyranoside (9a)**

Compound **9a** was prepared in a similar manner as **6a** from 1,2,3,4,6-penta-*O*-acetyl-β-D-glucopyranose (500 mg, 1.28 mmol), 4-methylphenol (190 mg, 1.79 mmol) and boron trifluoride diethyl etherate (800 μL, 6.40 mmol) with 4 Å MS in anhydrous CH_2Cl_2 (6 mL) at 0 °C. Column chromatography (7:3 hexanes/EtOAc) afforded **9a** as a white powder (205 mg, 37%). Characterization data is consistent with that previously reported in the literature[11]. ^{1}H NMR (400 MHz, $CDCl_3$): δ 7.09 (d, *J* = 8.2 Hz, 2H), 6.89 (d, *J* = 8.6 Hz, 2H), 5.31-5.23 (m, 2H), 5.16 (t, *J* = 9.6 Hz, 1H), 5.02 (d, *J* = 7.7 Hz, 1H), 4.29 (dd, *J* = 12.2, 5.3 Hz, 1H), 4.16 (dd, *J* = 12.3, 2.3 Hz, 1H), 3.85-3.81 (m, 1H), 2.30 (s, 3H), 2.08 (s, 3H), 2.06 (s, 3H), 2.05 (s, 3H), 2.03 (s, 3H). ^{13}C NMR (101 MHz, $CDCl_3$): δ 170.6, 170.3, 169.4, 169.3, 154.8, 132.9, 130.0, 117.0, 99.6, 72.8, 72.0, 71.2, 68.3, 62.0, 20.7, 20.7, 20.6, 20.6, 20.6. LRMS (ESI): *m/z* calcd. for $C_{21}H_{30}NO_{10}$ $[M+NH_4]^+$ 456.4; found, 456.1.

***p*-Methylphenyl-β-D-glucopyranoside (9)**

Compound **9a** (124 mg, 0.28 mmol) was dissolved in a solution of sodium methoxide in methanol (5 mL) and stirred for one hour at room temperature. The solution was then neutralized with Amberlite® IR-120 (H^+) ion-exchange resin, filtered and concentrated. The product was purified by recrystallization in hot ethyl acetate to afford **9** as a white powder (68 mg, 89%). Characterization data is consistent with that previously reported in the literature[11]. ^{1}H NMR (400 MHz, D_2O): δ 7.24-7.22 (m, 2H), 7.06-7.04 (m, 2H), 5.08 (d, *J* = 7.6 Hz, 1H), 3.92 (dd,

J = 12.4, 2.3 Hz, 1H), 3.75 (dd, J = 12.4, 5.7 Hz, 1H), 3.63-3.53 (m, 3H), 3.49 (dd, J = 10.0, 8.6 Hz, 1H), 2.30 (s, 3H). ^{13}C NMR (101 MHz, D_2O): δ 154.4, 133.3, 130.2, 116.6, 100.5, 76.1, 75.6, 73.0, 69.5, 60.6, 19.6. LRMS (ESI): *m/z* calcd. for $C_{13}H_{18}NaO_6$ $[M+Na]^+$ 293.2; found, 293.1.

Phenyl-2,3,4,6-tetra-*O*-acetyl-β-D-glucopyranoside (10a)

Compound **10a** was prepared in a similar manner as **6a** from 1,2,3,4,6-penta-*O*-acetyl-β-D-glucopyranose (500 mg, 1.28 mmol), phenol (169 mg, 1.79 mmol) and boron trifluoride diethyl etherate (209 μL, 1.66 mmol) with 4 Å MS in anhydrous CH_2Cl_2 (10 mL) at 0 °C. Column chromatography (7:3 hexanes/EtOAc) afforded **10a** as a white powder (420 mg, 77%). Characterization data is consistent with that previously reported in the literature[2]. ^{1}H NMR (400 MHz, $CDCl_3$): δ 7.30 (dd, J = 8.7, 7.4 Hz, 2H), 7.08 (dd, J = 7.8, 7.0 Hz, 1H), 6.99 (dt, J = 7.8, 1.0 Hz, 2H), 5.33-5.25 (m, 2H), 5.17 (t, J = 9.7 Hz, 1H), 5.09 (d, J = 7.8 Hz, 1H), 4.29 (dd, J = 12.3, 5.4 Hz, 1H), 4.17 (dd, J = 12.3, 2.5 Hz, 1H), 3.86 (ddd, J = 10.0, 5.3, 2.5 Hz, 1H), 2.09 (s, 3H), 2.06 (s, 3H), 2.05 (s, 3H), 2.04 (s, 3H).^{13}C NMR (75 MHz, $CDCl_3$): δ 170.6, 170.2, 169.4, 169.3, 156.8, 129.6, 123.4, 117.0, 99.1, 72.7, 72.0, 71.2, 68.3, 61.9, 20.7, 20.6, 20.6. LRMS (ESI): *m/z* calcd. for $C_{20}H_{23}O_{10}$ $[M-H]^-$ 423.4; found, 423.3.

Phenyl-β-D-glucopyranoside (10)

Compound **10a** (400 mg, 0.94 mmol) was dissolved in a solution of sodium methoxide in methanol (5 mL) and stirred for one hour at room temperature. The solution was then neutralized with Amberlite® IR-120 (H^+) ion-exchange resin, filtered and concentrated. The filtrate was concentrated and the product was lyophilized to yield **10** as a white powder (229 mg, 95%). ^{1}H NMR (400 MHz, D_2O): δ 7.41-7.37 (m, 2H), 7.16-7.13 (m, 3H), 5.13 (d, J = 7.5 Hz, 1H), 3.94-3.90 (m, 1H), 3.74 (dd, J = 12.5, 5.7 Hz, 1H), 3.64-3.54 (m, 3H), 3.48 (dd, J = 9.8, 8.9 Hz, 1H). ^{13}C NMR (101 MHz, D_2O): δ 156.5, 129.9, 123.3, 116.5, 100.1, 76.1, 75.5, 72.9, 69.4, 60.5 LRMS (ESI): *m/z* calcd. for $C_{12}H_{15}O_6$ $[M-H]^-$ 255.3; found, 255.1.

***p*-Nitrophenyl-2,3,4,6-tetra-*O*-acetyl-β-D-glucopyranoside (11a)**

Compound **11a** was prepared in a similar manner as **6a** from 1,2,3,4,6-Penta-*O*-acetyl-β-D-glucopyranose (420 mg, 1.08 mmol), 4-nitrophenol (210 mg, 1.51 mmol) and boron trifluoride diethyl etherate (180 μL, 1.40 mmol) with 4 Å MS in anhydrous CH_2Cl_2 (10 mL) at 0 °C. Column chromatography (8:2 hexanes/EtOAc) afforded **11a** as a white powder (347 mg, 68%). Characterization data is consistent with that previously reported in the literature[2]. ^{1}H NMR (500 MHz, $CDCl_3$): δ 8.23-8.20 (m, 2H), 7.09-7.06 (m, 2H), 5.16-5.11 (m, 3H), 5.09 (d, J = 3.7 Hz, 1H), 4.28 (dd, J = 12.3, 5.2 Hz, 1H), 4.18 (dd, J = 12.3, 2.3 Hz, 1H), 3.86-3.82 (m, 1H), 2.07 (s, 3H), 2.06 (s, 3H), 2.03 (s, 3H), 2.02 (s, 3H). ^{13}C NMR (101 MHz, $CDCl_3$): δ 170.7, 170.2, 169.7, 169.3, 160.8, 140.9, 124.7, 115.5, 98.1, 74.7, 73.9, 71.2, 68.1, 61.9, 20.9, 20.7, 20.6, 20.5. LRMS (ESI): *m/z* calcd. for $C_{20}H_{22}NO_{12}$ $[M-H]^-$ 468.4; found, 468.2.

***p*-Nitrophenyl -β-D-glucopyranoside (11)**

Compound **11a** (250 mg, 0.53 mmol) was dissolved in a solution of sodium methoxide in methanol (4 mL) and stirred for one hour at room temperature. The solution was then neutralized with Amberlite® IR-120 (H^+) ion-exchange resin, filtered and concentrated. The product was purified by column chromatography (4:1:1:1 EtOAc/ACN/H_2O/MeOH) to afford **11** as a white powder (128 mg, 80%). ^1H NMR (400 MHz, D_2O): δ 8.28-8.24 (m, 2H), 7.26-7.22 (m, 2H), 5.27 (d, *J* = 7.7 Hz, 1H), 3.93 (dd, *J* = 12.3, 2.1 Hz, 1H), 3.75 (dd, *J* = 12.3, 5.7 Hz, 1H), 3.71-3.67 (m, 1H), 3.63-3.61 (m, 2H), 3.51 (t, *J* = 9.4 Hz, 1H). ^{13}C NMR (101 MHz, D_2O): δ 161.7, 142.6, 126.1, 116.4, 99.4, 76.3, 75.4, 72.7, 69.3, 60.4. LRMS (ESI): *m/z* calcd. for $C_{12}H_{14}NO_8$ $[M-H]^-$ 300.3; found, 300.5. LRMS (ESI): *m/z* calcd. for $C_{24}H_{28}N_2O_{16}$ $[M-H]^-$ Dimer 601.5; found, 601.0.

***p*-Fluorophenyl-2,3,4,6-tetra-*O*-acetyl-β-D-glucopyranoside (12a)**

Compound **12a** was prepared in a similar manner as **6a** from 1,2,3,4,6-penta-*O*-acetyl-β-D-glucopyranose (500 mg, 1.28 mmol), 4-fluorophenol (200 mg, 1.58 mmol) and boron trifluoride diethyl etherate (803 μL, 6.4 mmol) with 4 Å MS in anhydrous CH_2Cl_2 (6 mL) at 0 °C. Column chromatography (3:2 hexanes/ethyl acetate) afforded **12a** as a white powder (416 mg, 77%). Characterization data is consistent with that previously reported[12]. ^1H NMR (400 MHz, $CDCl_3$): δ 7.10-7.08 (m, 2H), 6.91-6.88 (m, 2H), 5.30-5.25 (m, 2H), 5.16 (t, *J* = 9.7 Hz, 1H), 5.03-5.01 (m, 1H), 4.29 (dd, *J* = 12.2, 5.2 Hz, 1H), 4.17 (dd, *J* = 12.2, 2.5 Hz, 1H), 3.83 (ddd, *J* = 10.0, 5.3, 2.5 Hz, 1H), 2.08 (s, 3H), 2.06 (s, 3H), 2.04 (s, 3H), 2.03 (s, 3H). ^{13}C NMR (101 MHz, $CDCl_3$): δ 170.5, 170.2, 169.4, 169.2, 160.0, 157.6, 152.9, 152.9, 118.8, 118.7, 116.1, 115.9, 99.8, 72.6, 72.1, 71.2, 68.2, 61.9, 20.7, 20.6, 20.6, 20.6. LRMS (ESI): *m/z* calcd for $C_{20}H_{27}FNO_{10}$ $[M+NH_4]^+$ 460.5; found, 460.2.

***p*-Fluorophenyl-β-D-glucopyranoside (12)**

Compound **12a** (198 mg, 0.45 mmol) was dissolved in a solution of sodium methoxide in methanol (5 mL) and stirred for one hour at room temperature. The solution was then neutralized with Amberlite® IR-120 (H^+) ion-exchange resin, filtered and concentrated. The filtrate was concentrated and the product was lyophilized to yield **12** as a white powder (120 mg, 98%). ^1H NMR (400 MHz, D_2O): δ 7.15-7.09 (m, 4H), 5.06 (d, *J* = 7.5 Hz, 1H), 3.93 (dd, *J* = 12.4, 2.3 Hz, 1H), 3.75 (dd, *J* = 12.4, 5.6 Hz, 1H), 3.63-3.53 (m, 3H), 3.52-3.47 (m, 1H). ^{13}C NMR (101 MHz, D_2O): δ 152.7, 118.2, 118.2, 116.2, 116.0, 100.9, 76.1, 75.5, 72.9, 69.4, 60.5. LRMS (ESI): *m/z* calcd. for $C_{12}H_{15}FNaO_6$ $[M+Na]^+$ 297.2; found, 297.1.

***p*-Bromophenyl-2,3,4,6-tetra-*O*-acetyl-β-D-glucopyranoside (13a)**

Compound **13a** was prepared in a similar manner as **6a** from 1,2,3,4,6-penta-*O*-acetyl-β-D-glucopyranose (1.5 g, 3.84 mmol), 4-bromophenol (798 mg, 4.61 mmol) and boron trifluoride diethyl etherate (723 μL, 5.76 mmol) with

4 Å MS in anhydrous CH_2Cl_2 (15 mL) at 0 °C. Column chromatography (4:1 hexanes/ethyl acetate) afforded **13a** as a white powder (1.33 g, 69%). Characterization data is consistent with that previously reported[13]. ^{1}H NMR (400 MHz, $CDCl_3$): δ 7.42-7.39 (m, 2H), 6.90-6.86 (m, 2H), 5.31-5.23 (m, 2H), 5.16 (t, *J* = 9.6 Hz, 1H), 5.03 (d, *J* = 7.6 Hz, 1H), 4.28 (dd, *J* = 12.3, 5.4 Hz, 1H), 4.16 (dd, *J* = 12.3, 2.4 Hz, 1H), 3.84 (ddd, *J* = 10.0, 5.4, 2.5 Hz, 1H), 2.08 (s, 3H), 2.06 (s, 3H), 2.05 (s, 3H), 2.03 (s, 3H). ^{13}C NMR (101 MHz, $CDCl_3$): δ 170.5, 170.2, 169.4, 155.8, 132.5, 118.8, 115.9, 99.1, 72.6, 72.1, 71.1, 68.2, 61.9, 20.8, 20.8, 20.7, 20.6. LRMS (ESI): *m/z* calcd. for $C_{20}H_{27}BrNO_{10}$ $[M+NH_4]^+$ 520.2; found, 520.0.

***p*-Bromophenyl-β-D-glucopyranoside (13)**

Compound **13a** (200 mg, 0.40 mmol) was dissolved in a solution of sodium methoxide in methanol (5 mL) and stirred for one hour at room temperature. The solution was then neutralized with Amberlite® IR-120 (H^+) ion-exchange resin, filtered and concentrated. The filtrate was concentrated and the product was lyophilized to yield **13** as a white powder (123 mg, 92%). ^{1}H NMR (400 MHz, D_2O): δ 7.54-7.50 (m, 2H), 7.07-7.03 (m, 2H), 5.09 (d, *J* = 7.5 Hz, 1H), 3.92 (dd, *J* = 12.5, 2.3 Hz, 1H), 3.74 (dd, *J* = 12.5, 5.7 Hz, 1H), 3.64-3.53 (m, 3H), 3.49 (dd, *J* = 9.7, 8.9 Hz, 1H). ^{13}C NMR (101 MHz, D_2O): δ 155.7, 132.6, 118.5, 115.0, 100.2, 76.1, 75.5, 72.9, 69.4, 60.5. LRMS (ESI): *m/z* calcd. for $C_{12}H_{15}BrNaO_6$ $[M+Na]^+$ 357.0; found, 357.0.

***m*-Methoxyphenyl-2,3,4,6-tetra-*O*-acetyl-β-D-glucopyranoside (14a)**

Compound **14a** was prepared in a similar manner as **6a** from 1,2,3,4,6-penta-*O*-acetyl-β-D-glucopyranose (500 mg, 1.28 mmol), 3-methoxyphenol (222 mg, 1.79 mmol) and boron trifluoride diethyl etherate (803 μL, 6.4 mmol) with 4 Å MS in anhydrous CH_2Cl_2 (10 mL) at 0 °C. Column chromatography (3:2 hexanes/EtOAc) afforded **14a** as a white powder (162 mg, 28%). ^{1}H NMR (400 MHz, $CDCl_3$): δ 7.21-7.17 (m, 1H), 6.64-6.56 (m, 3H), 5.32-5.24 (m, 2H), 5.16 (t, *J* = 9.7 Hz, 1H), 5.08 (d, *J* = 7.7 Hz, 1H), 4.28 (dd, *J* = 12.3, 5.5 Hz, 1H), 4.17 (dd, *J* = 12.2, 2.4 Hz, 1H), 3.86 (ddd, *J* = 10.0, 5.5, 2.5 Hz, 1H), 3.78 (s, 3H), 2.08 (s, 3H), 2.06 (s, 3H), 2.05 (s, 3H), 2.03 (s, 3H). ^{13}C NMR (101 MHz, $CDCl_3$): δ 170.6, 170.2, 169.4, 169.3, 160.8, 158.0, 130.0, 108.9, 108.6, 103.6, 99.0, 72.7, 72.0, 71.1, 68.3, 62.0, 55.4, 30.9, 20.6, 20.6, 20.6. LRMS (ESI): *m/z* calcd. for $C_{21}H_{30}NO_{11}$ $[M+NH_4]^+$ 475.5; found, 472.1.

***m*-Methoxyphenyl-β-D-glucopyranoside (14)**

Compound **14a** (162 mg, 0.33 mmol) was dissolved in a solution of sodium methoxide in methanol (5 mL) and stirred for one hour at room temperature. The solution was then neutralized with Amberlite® IR-120 (H^+) ion-exchange resin, filtered and concentrated. The filtrate was concentrated and the product was lyophilized to yield **14** as a white powder (90 mg, 95%). ^{1}H NMR (400 MHz, D_2O): δ 7.34-7.30 (m, 1H), 6.78-6.75 (m, 3H), 5.12 (d, *J* =

7.2 Hz, 1H), 3.93 (dd, J = 12.1, 1.2 Hz, 1H), 3.82 (s, 3H), 3.76-3.72 (m, 1H), 3.65-3.54 (m, 3H), 3.51-3.46 (m, 1H). ^{13}C NMR (101 MHz, D_2O): δ 160.1, 157.7, 130.6, 109.0, 108.9, 102.9, 100.1, 76.2, 75.5, 72.9, 69.5, 60.6, 55.5. LRMS (ESI): *m/z* calcd. for $C_{13}H_{22}NO_7$ $[M+NH_4]^+$ 304.4; found, 304.1.

Synthesis of p-methoxybenzyl-glucopyranoside 15:

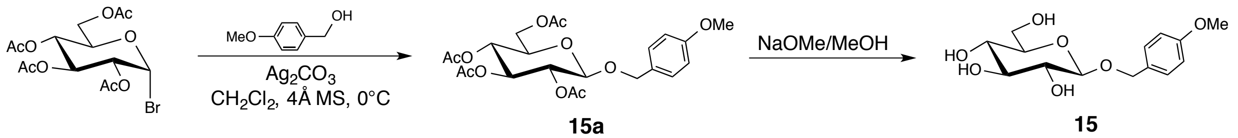

Figure S6. Synthesis of β-PMB-Glc (**15**).

***p*-Methoxybenzyl-2,3,4,6-tetra-*O*-acetyl-β-D-glucopyranoside (15a)**

To a mixture of 4-methoxybenzyl alcohol (109 mg, 0.79 mmol), Ag_2CO_3 (250 mg, 0.91 mmol) and 4 Å MS in anhydrous CH_2Cl_2 (3 mL) stirring in the dark under Ar was added bromo-2,3,4,6-tetra-*O*-acetyl-β-D-glucopyranose[14] (250 mg, 0.61 mmol) in anhydrous CH_2Cl_2 (2 mL) dropwise over 20 minutes. The reaction mixture was diluted with CH_2Cl_2 and filtered through Celite®, then washed with sodium bicarbonate, water, saturated brine, then dried over $MgSO_4$ and concentrated. Flash column chromatography over a gradient (8:2 hexanes/EtOAc to 3:2 hexanes/EtOAc) afforded **15a** as a white solid (88 mg, 31%). ^{1}H NMR (400 MHz, $CDCl_3$): δ 7.22-7.19 (m, 2H), 6.89-6.85 (m, 2H), 5.18-5.07 (m, 2H), 5.03 (dd, J = 9.5, 8.0 Hz, 1H), 4.82 (d, J = 11.9 Hz, 1H), 4.56 (d, J = 11.9 Hz, 1H), 4.51 (d, J = 7.9 Hz, 1H), 4.27 (dd, J = 12.3, 4.7 Hz, 1H), 4.17 (dd, J = 12.3, 2.4 Hz, 1H), 3.81 (s, 3H), 3.66 (ddd, J = 9.7, 4.7, 2.5 Hz, 1H), 2.11 (s, 3H), 2.01 (s, 3H), 1.99 (s, 3H), 1.99 (s, 3H). ^{13}C NMR (101 MHz, $CDCl_3$): δ 170.7, 170.3, 169.4, 169.3, 159.5, 129.5, 128.5, 113.9, 98.8, 72.8, 71.8, 71.3, 70.4, 68.4, 62.0, 55.3, 20.8, 20.7, 20.6, 20.6. LRMS (ESI): *m/z* calcd. for $C_{22}H_{32}NO_{11}$ $[M+NH_4]^+$ 486.5; found, 486.1.

***p*-Methoxybenzyl-β-D-glucopyranoside (15)**

Compound **15a** (70 mg, 0.15 mmol) was dissolved in a solution of sodium methoxide in methanol (3 mL) and stirred for one hour at room temperature. The solution was then neutralized with Amberlite® IR-120 (H^+) ion-exchange resin, filtered and concentrated. The product was purified by recrystallization in hot ethyl acetate to afford **15** as a white powder (34 mg, 76%). ^{1}H NMR (400 MHz, D_2O): δ 7.44-7.41 (m, 2H), 7.05-7.01 (m, 2H), 4.88 (d, J = 11.3 Hz, 1H), 4.70 (d, J = 11.4 Hz, 1H), 4.50 (d, J = 8.0 Hz, 1H), 3.92 (dd, J = 12.3, 2.0 Hz, 1H), 3.85 (s, 3H), 3.73 (dd, J = 12.3, 5.7 Hz, 1H), 3.47-3.38 (m, 3H), 3.28 (dd, J = 9.0, 8.1 Hz, 1H). ^{13}C NMR (101 MHz,

D_2O): δ 158.9, 130.6, 129.1, 114.1, 100.9, 75.92, 75.82, 73.1, 71.1, 69.7, 60.8, 55.4. LRMS (ESI): *m/z* calcd. for $C_{14}H_{24}NO_7$ $[M+NH_4]^+$ 318.3; found, 318.2.

Synthesis of α–linked p-methoxyphenyl-glucopyranoside 16:

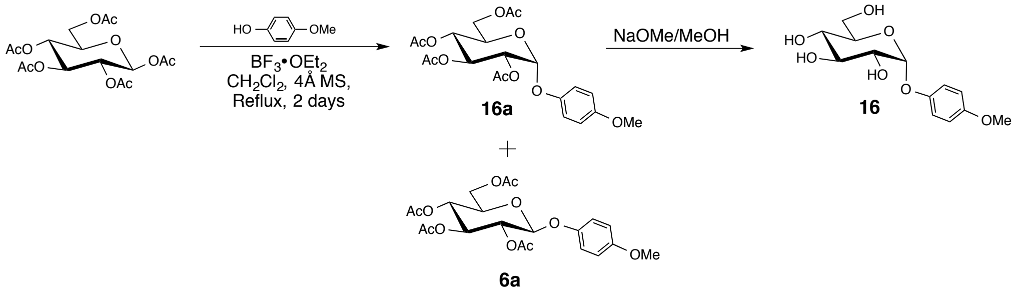

Figure S7. Synthesis of α-PMP-Glc (**16**).

***p*-Methoxyphenyl-2,3,4,6-tetra-*O*-acetyl-α-D-glucopyranoside (16a)**

Compound **16a** was prepared in a similar manner as **6a** from 1,2,3,4,6-penta-*O*-acetyl-β-D-glucopyranose (4 g, 10.3 mmol), 4-methoxyphenol (1.78 g, 14.4 mmol) and boron trifluoride diethyl etherate (2.6 mL, 20.5 mmol) with 4 Å MS in anhydrous CH_2Cl_2 (60 mL). The reaction mixture was refluxed for 2 days, and following quenching, washing and concentration the crude mixture showed a 1:1.6 ratio of α:β-linked products (**16a**:**6a**). Column chromatography (4:1 hexanes/EtOAc) afforded **16a** as a white powder (1.43 g, 30%). Characterization data is consistent with that previously reported in the literature[15]. ^{1}H NMR (300 MHz, $CDCl_3$): δ 7.02-6.97 (m, 2H), 6.84-6.79 (m, 2H), 5.68 (dd, *J* = 10.0, 9.7 Hz, 1H), 5.61 (d, *J* = 3.6 Hz, 1H), 5.13 (dd, *J* = 10.1, 9.4 Hz, 1H), 5.00 (dd, *J* = 10.3, 3.7 Hz, 1H), 4.24 (dd, *J* = 12.1, 4.5 Hz, 1H), 4.15 (ddd, *J* = 10.2, 4.5, 2.0 Hz, 1H), 4.06 (dd, *J* = 12.1, 2.2 Hz, 1H), 3.76 (s, 3H), 2.06 (s, 3H), 2.05 (s, 3H), 2.04 (s, 3H), 2.03 (s, 3H). ^{13}C NMR (101 MHz, $CDCl_3$): δ 170.6, 170.3, 169.4, 169.3, 158.7, 151.5, 118.7, 115.3, 96.2, 72.2, 71.4, 71.2, 68.3, 61.9, 55.8, 20.7, 20.7, 20.6, 20.6. LRMS (ESI): *m/z* calcd. for $C_{21}H_{30}NO_{11}$ $[M+NH_4]^+$ 472.5; found, 472.3.

***p*-Methoxyphenyl-2,3,4,6-tetra-*O*-acetyl-α-D-glucopyranoside (16)**

Compound **16a** (1.43 g, 3.15 mmol) was dissolved in a solution of sodium methoxide in methanol (15 mL) and stirred for one hour at room temperature. The solution was then neutralized with Amberlite® IR-120 (H^+) ion-exchange resin, filtered and concentrated. The filtrate was concentrated and the product was lyophilized to yield **16** as a white powder (780 mg, 87%). ^{1}H NMR (300 MHz, D_2O): δ 7.17-7.12 (m, 2H), 7.02-6.96 (m, 2H), 5.52 (d, *J* = 3.7 Hz, 1H), 3.90 (t, *J* = 9.4 Hz, 1H), 3.84-3.76 (m, 3H), 3.81 (s, 3H), 3.70 (dd, *J* = 9.8, 3.8 Hz, 1H), 3.51 (t, *J* = 9.5 Hz, 1H). ^{13}C NMR (126 MHz, D_2O): δ 154.5, 150.2, 118.8, 114.9, 98.0, 72.9, 72.3, 71.0, 69.2, 60.1, 55.6. LRMS (ESI): *m/z* calcd. for $C_{13}H_{17}O_7$ $[M-H]^-$ 285.3; found, 285.1.

References

1. McGill, N. W. & Williams, S. J. 2,6-disubstituted benzoates as neighboring groups for enhanced diastereoselectivity in β-galactosylation reactions: Synthesis of β-1,3-linked oligogalactosides related to arabinogalactan proteins. *J. Org. Chem.* **74**, 9388-9398 (2009).
2. Smits, E., Engberts, J. B. F. N., Kellogg, R. M. & van Doren, H. A. Reliable method for the synthesis of aryl β-D-glucopyranosides, using boron trifluoride-diethyl ether as catalyst. *J. Chem. Soc., Perkin Trans. 1*, 2873-2877 (1996).
3. Mukhopadhyay, B., Kartha, K. P. R., Russell, D. A. & Field, R. A. Streamlined synthesis of per-*O*-acetylated sugars, glycosyl iodides, or thioglycosides from unprotected reducing sugars. *J. Org. Chem.* **69**, 7758-7760 (2004).
4. Saksena, R., Zhang, J. & Kováč, P. Synthesis of 2-(trimethylsilyl)ethyl α-D-mannopyranosides revisited. *J. Carbohydr. Chem.* **21**, 453-470 (2002).
5. Garegg, P. J., Kvarnström, I., Niklasson, A., Niklasson, G. & Svensson, S. C. T. Partial substitution of thioglycosides by phase transfer catalyzed benzoylation and benzylation. *J. Carbohydr. Chem.* **12**, 933-953 (1993).
6. Crich, D. & Sun, S. Direct chemical synthesis of β-mannopyranosides and other glycosides via glycosyl triflates. *Tetrahedron* **54**, 8321-8348 (1998).
7. Crich, D. & Sun, S. Direct synthesis of β-mannopyranosides by the sulfoxide method. *J. Org. Chem.* **62**, 1198-1199 (1997).
8. Crich, D. & Sun, S. Formation of β-mannopyranosides of primary alcohols using the sulfoxide method. *J. Org. Chem.* **61**, 4506-4507 (1996).
9. Bubb, W. A. NMR spectroscopy in the study of carbohydrates: Characterizing the structural complexity. *Conc. Magn. Reson. A* **19A**, 1-19 (2003).
10. Li, Z.-J., Cai, L.-N. & Cai, M.-S. Studies on glycosides. X. An alternate method for highly stereoselective synthesis of alkyl-β-D-glucopyranosides. *Synth. Commun.* **22**, 2121-2124 (1992).
11. Desai, R. N. & Blackwell, L. F. Tempo-mediated regiospecific oxidation of glucosides to glucuronides. *Synlett* **2003**, 1981-1984 (2003).
12. Aich, U. & Loganathan, D. Stereoselective single-step synthesis and X-ray crystallographic investigation of acetylated aryl 1,2-trans glycopyranosides and aryl 1,2-cis C2-hydroxy-glycopyranosides. *Carbohydr. Res.* **341**, 19-28 (2006).
13. Schwizer, D. *et al.* Antagonists of the myelin-associated glycoprotein: A new class of tetrasaccharide mimics. *Biorg. Med. Chem.* **14**, 4944-4957 (2006).
14. Czechura, P., Tam, R. Y., Dimitrijevic, E., Murphy, A. V. & Ben, R. N. The importance of hydration for inhibiting ice recrystallization with *C*-linked antifreeze glycoproteins. *J. Am. Chem. Soc.* **130**, 2928-2929 (2008).
15. Alberti, A. *et al.* Reactivity of glucosyl radical in the presence of phenols. *Tetrahedron* **52**, 10241-10248 (1996).

Spectra

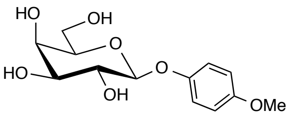

5
β-PMP-Gal

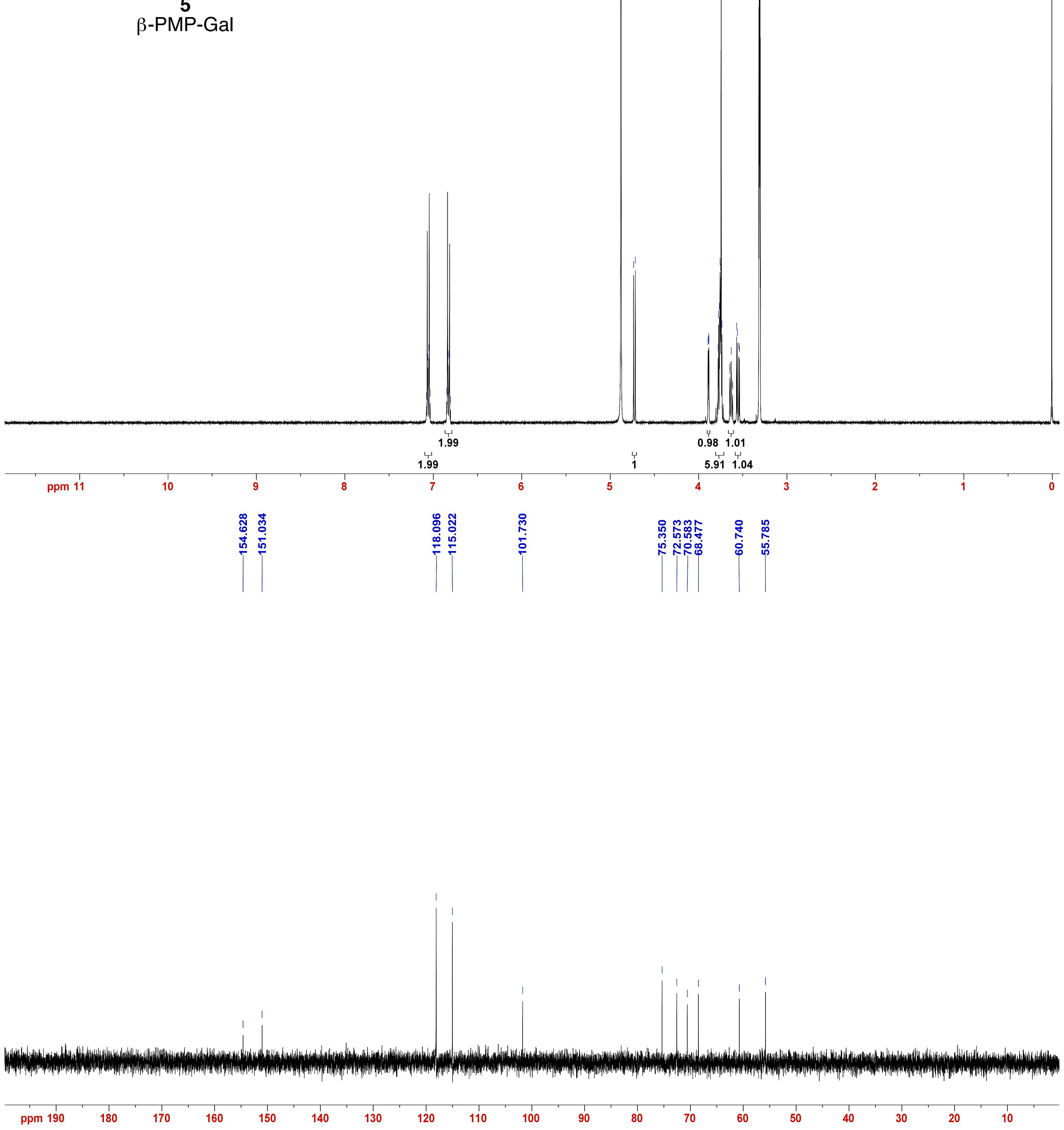

Figure S8. ^{1}H and ^{13}C spectra of β-PMP-Gal (**5**).

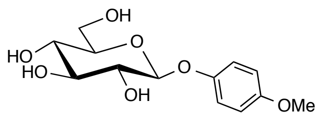

6
β-PMP-Glc

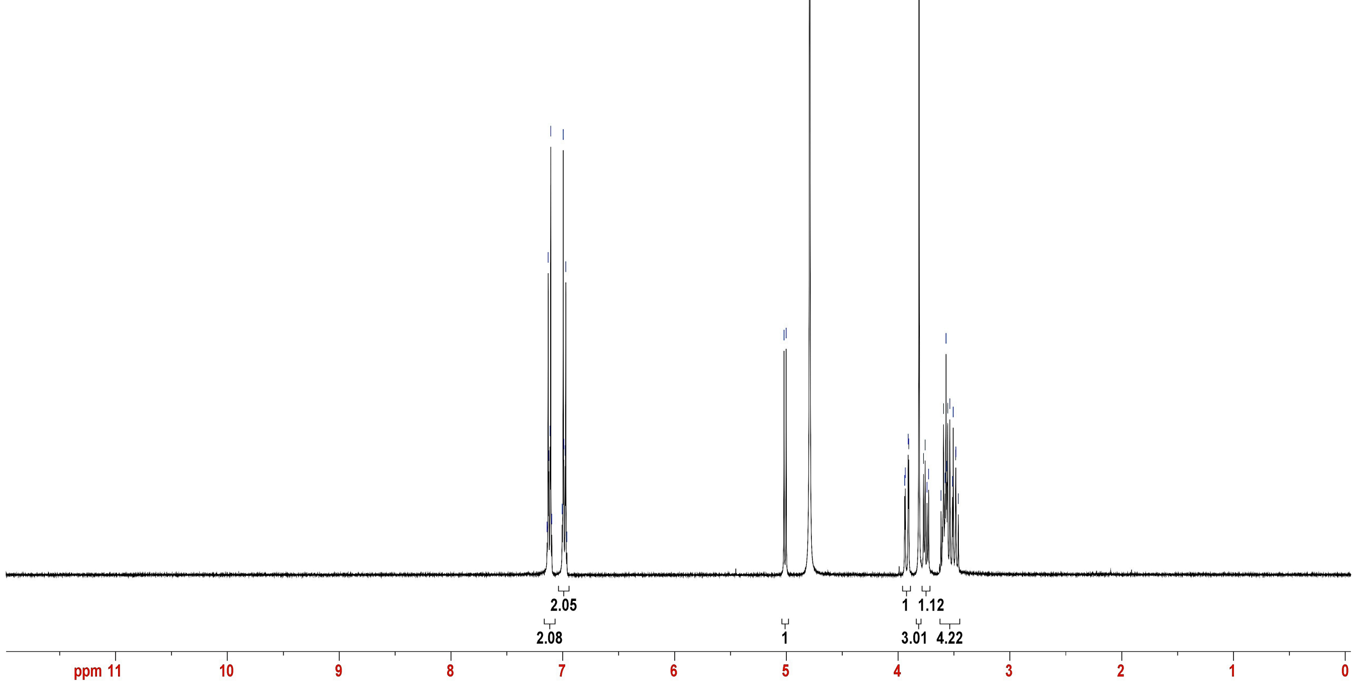

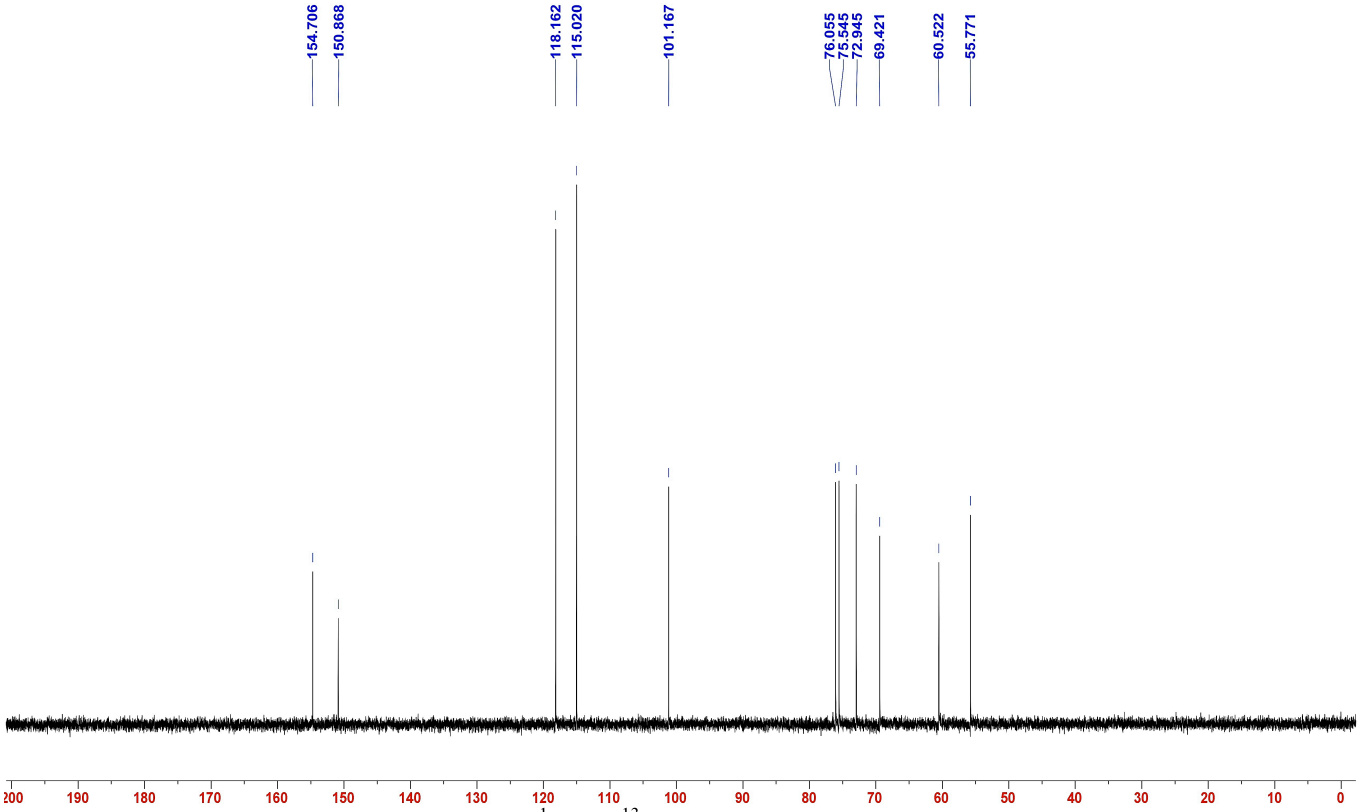

Figure S9. ^{1}H and ^{13}C spectra of β-PMP-Glc (**6**).

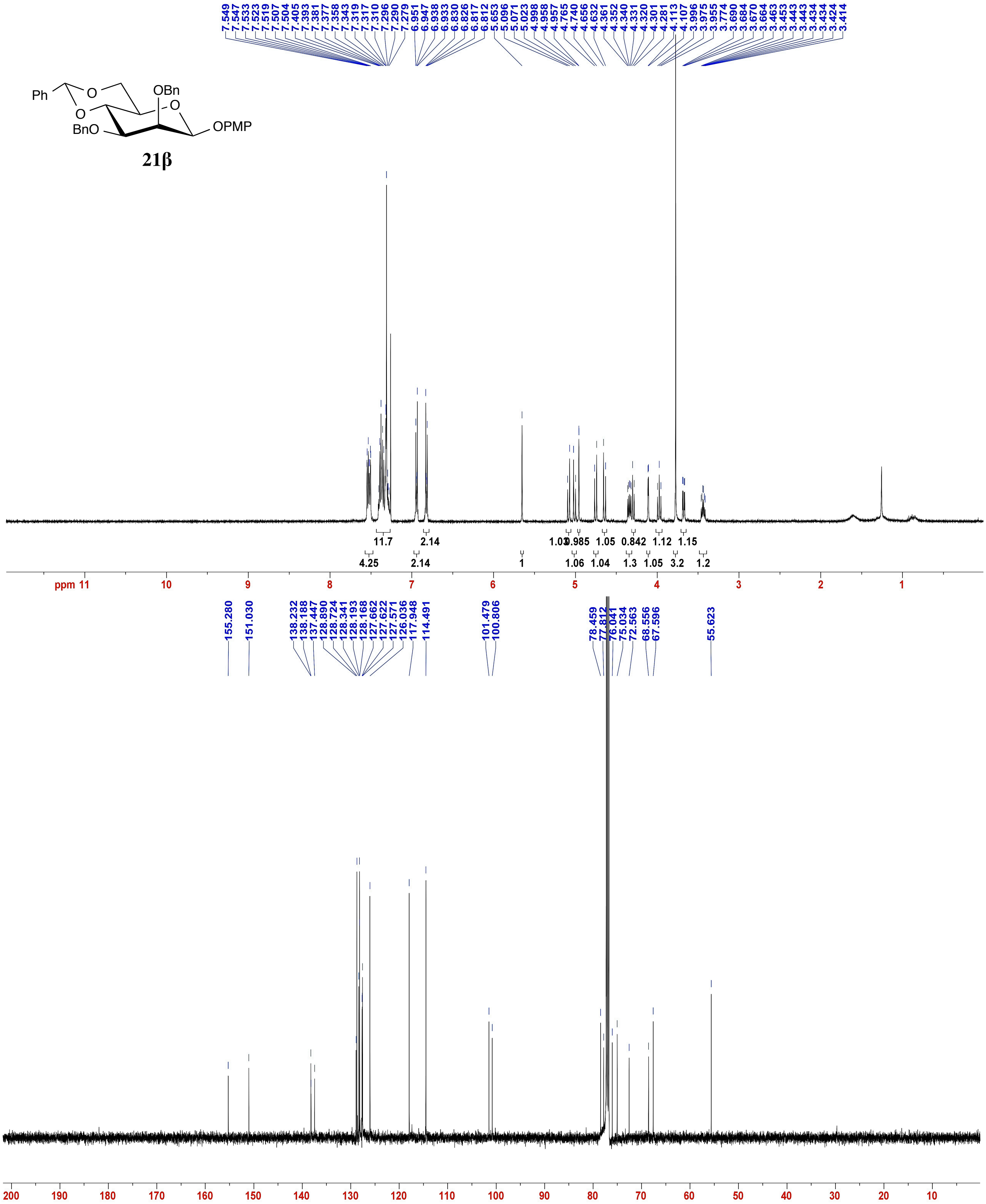

Figure S10. ^{1}H and ^{13}C spectra of **21β**.

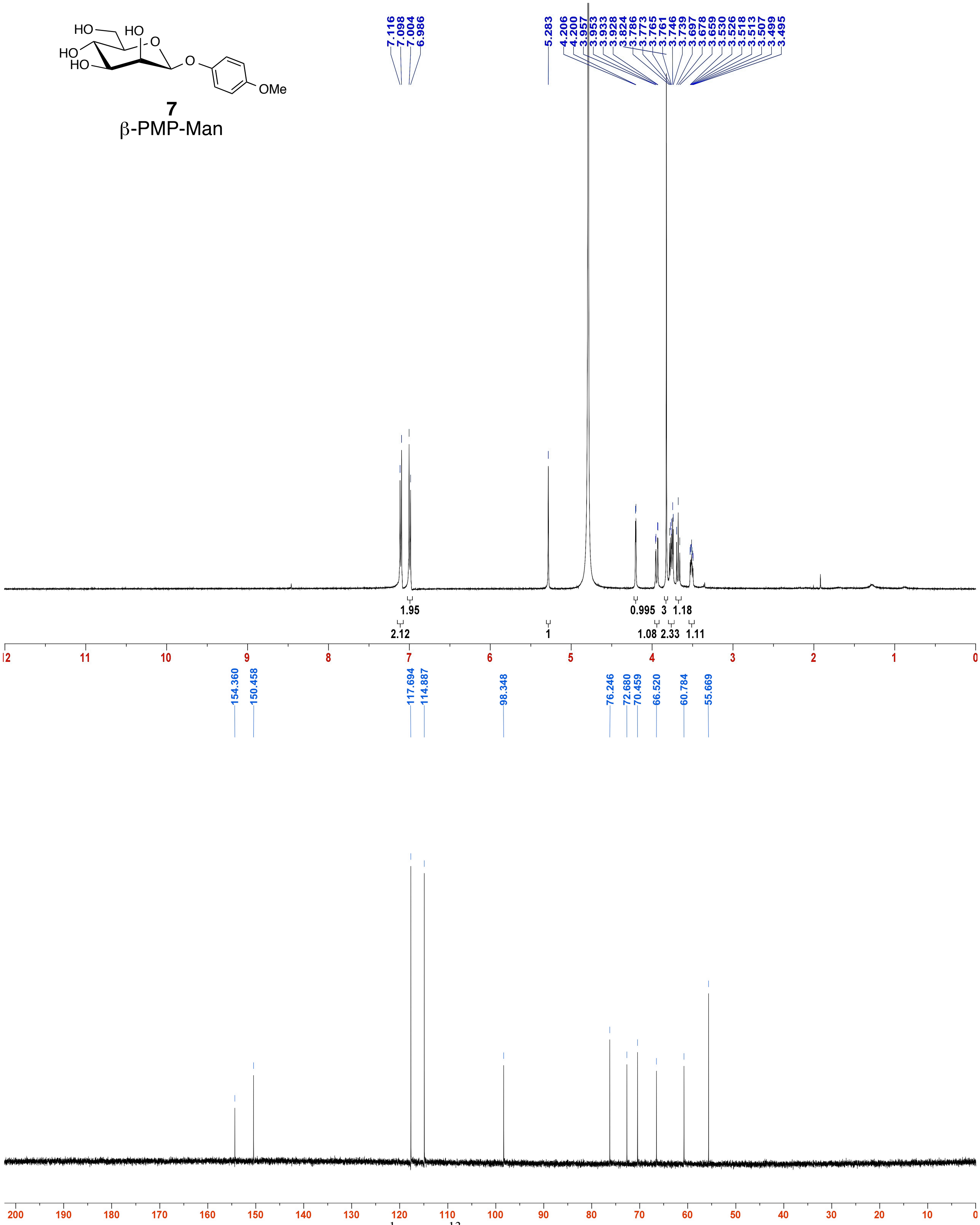

Figure S11. ^{1}H and ^{13}C spectra of β-PMP-Man (**7**).

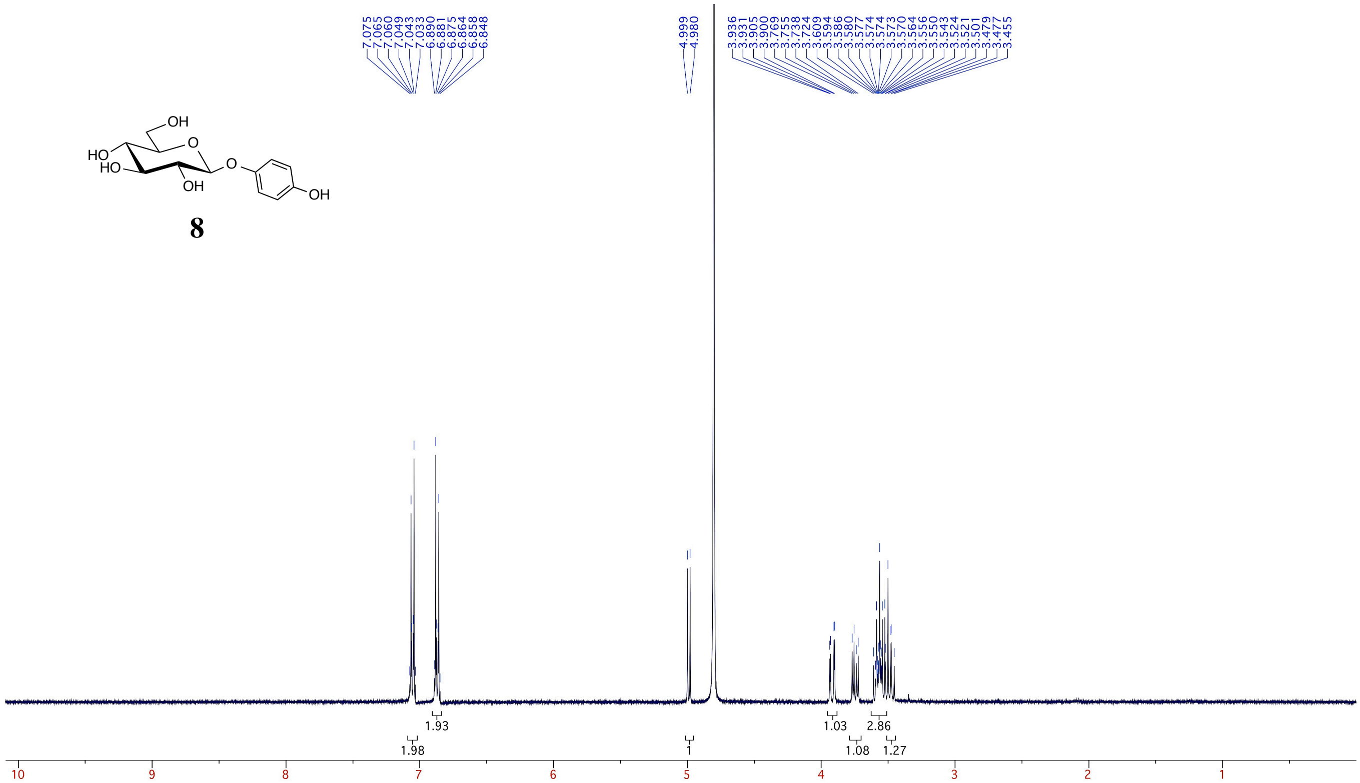

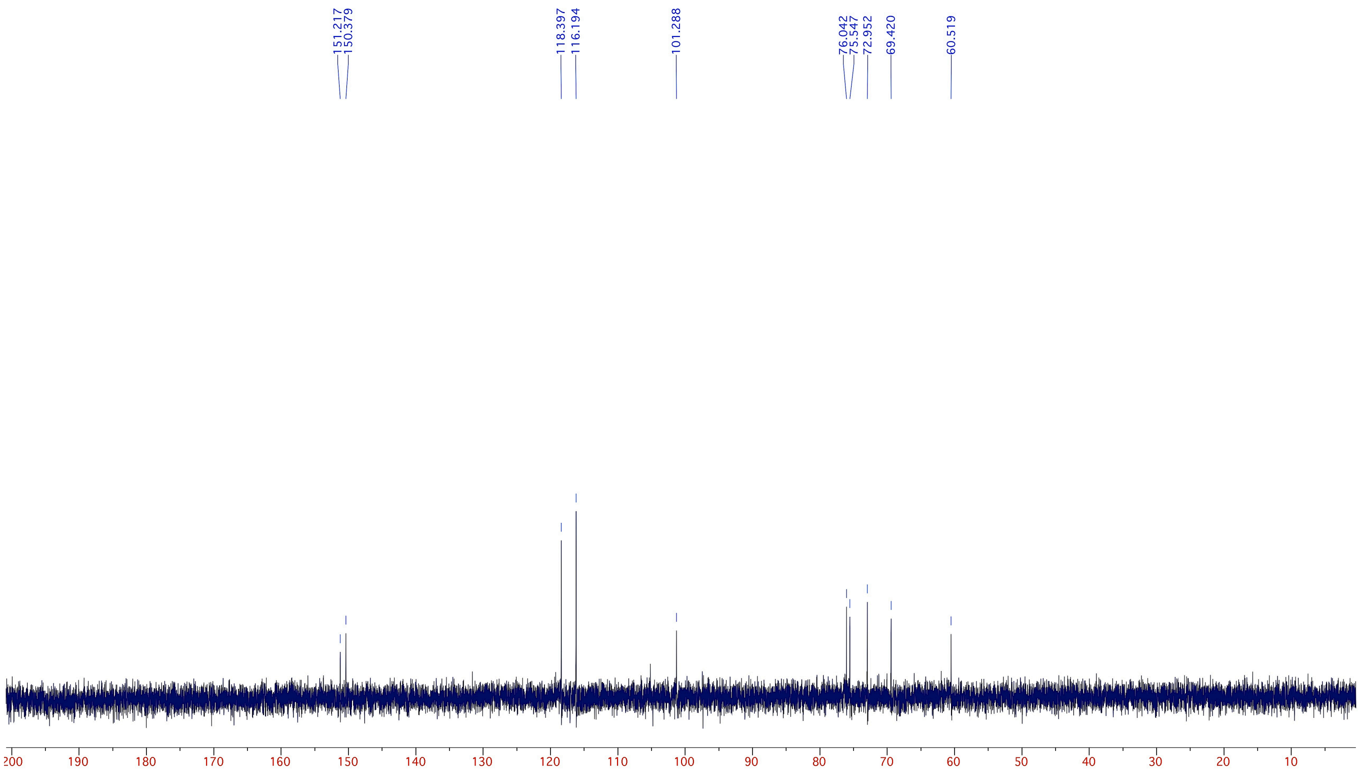

Figure S12. ^{1}H and ^{13}C spectra of β-pOHPh-Glc (**8**).

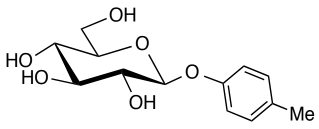

9

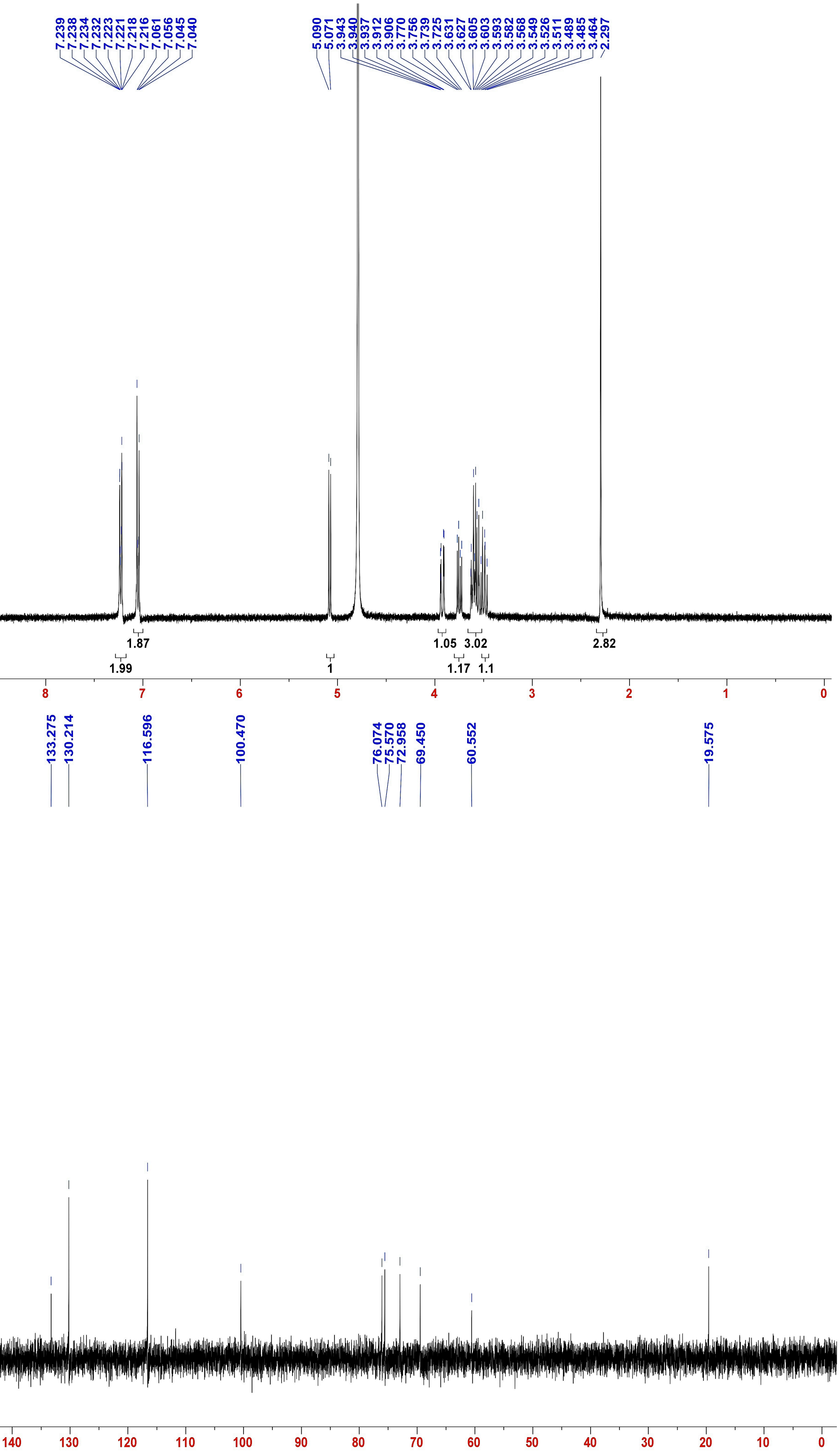

Figure S13. ^{1}H and ^{13}C spectra of β-pMePh-Glc (**9**).

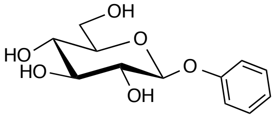

10

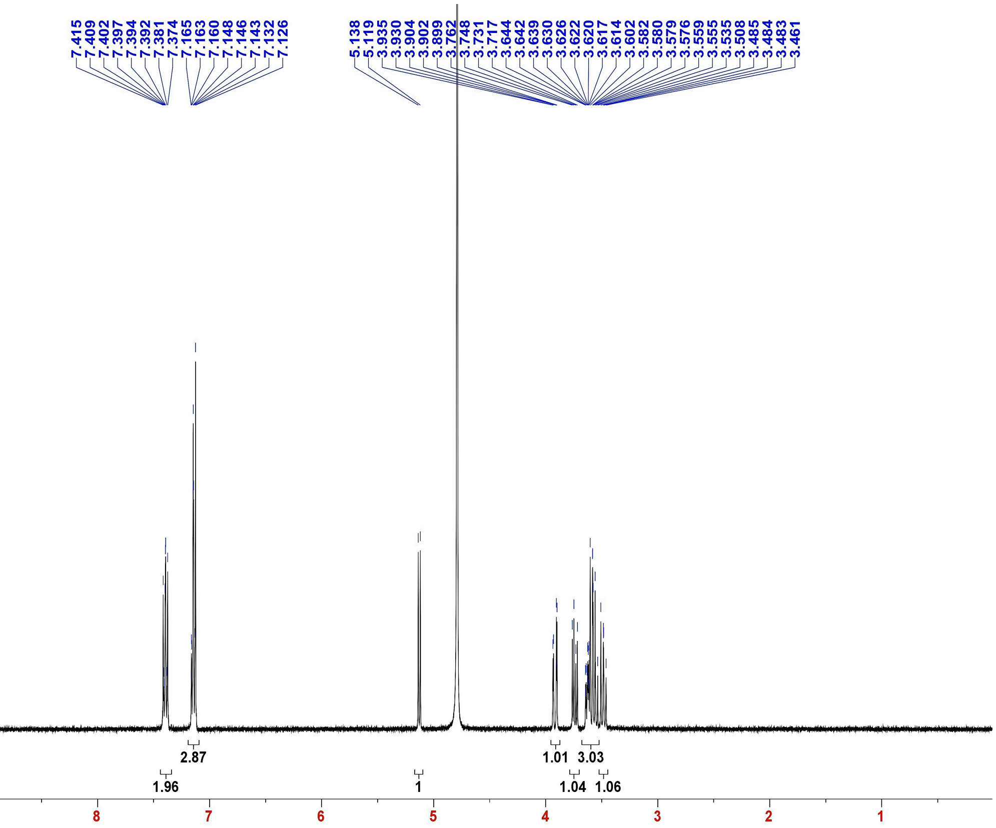

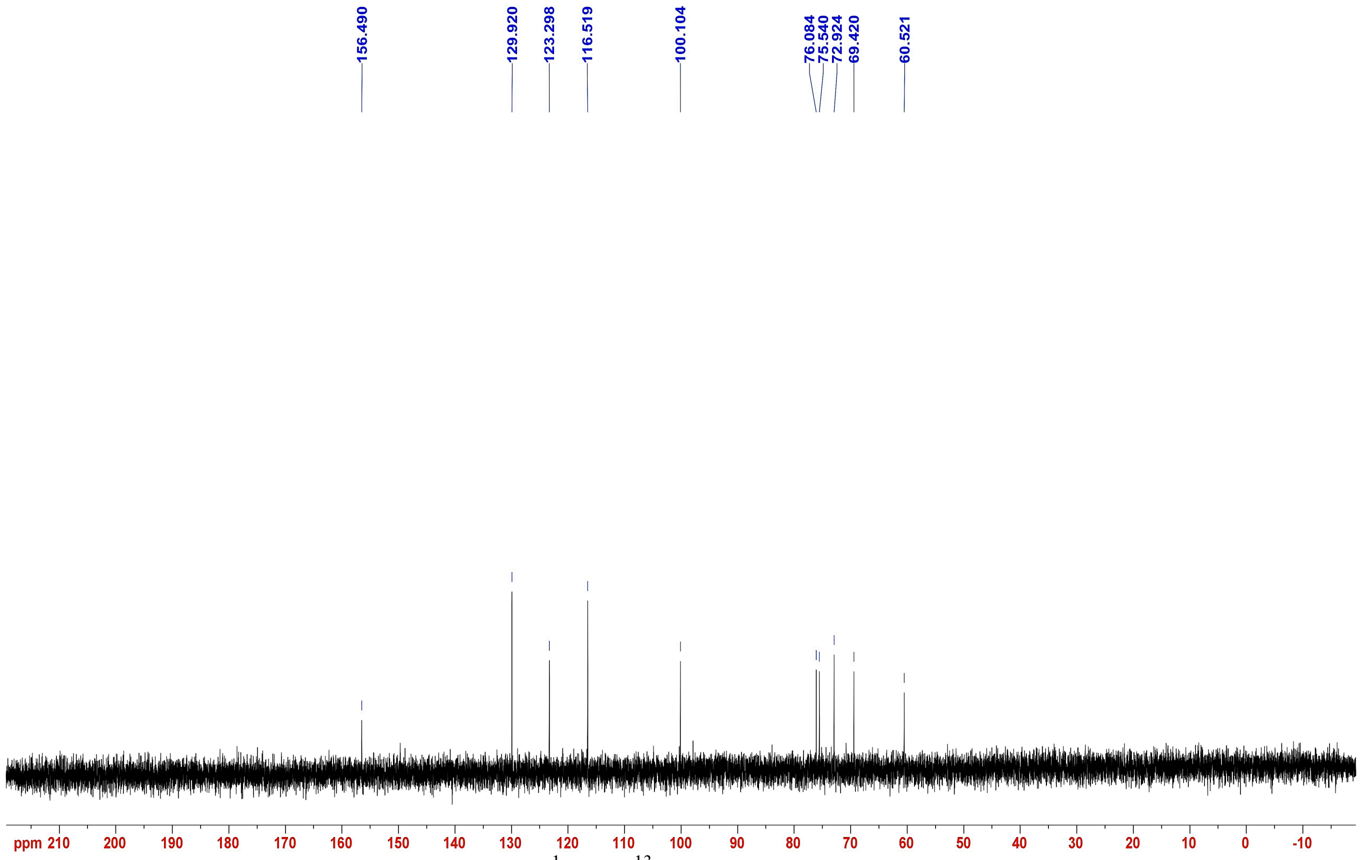

Figure S14. ^{1}H and ^{13}C spectra of β-Ph-Glc (**10**).

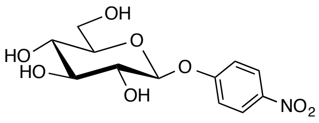

11

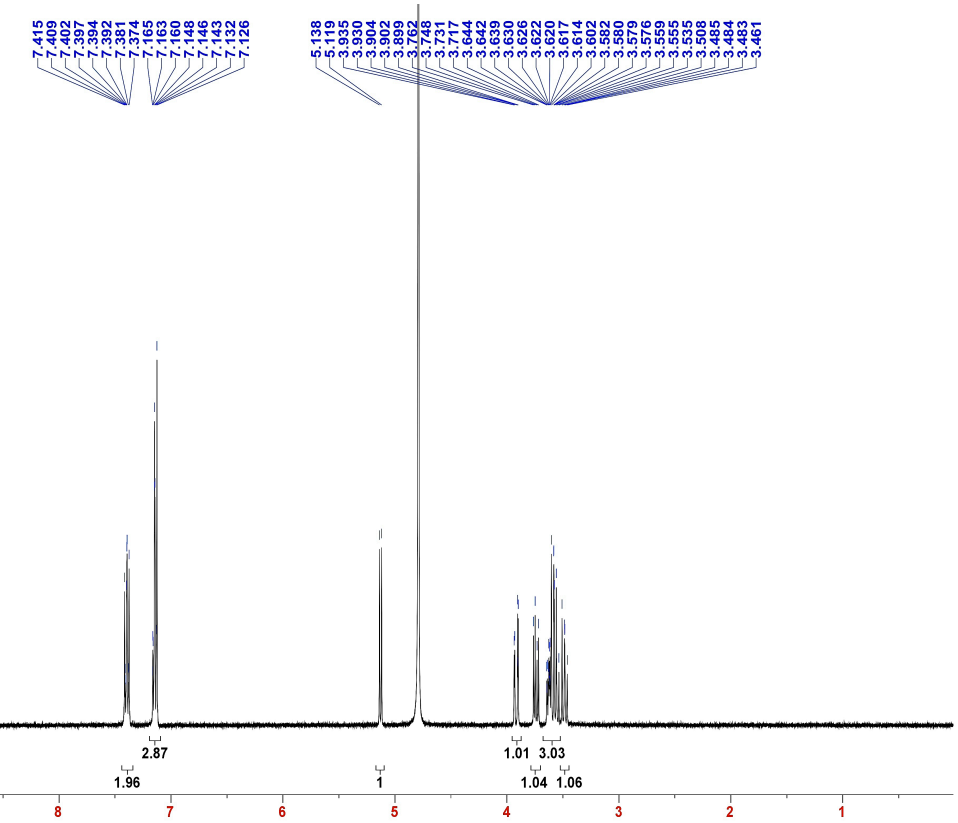

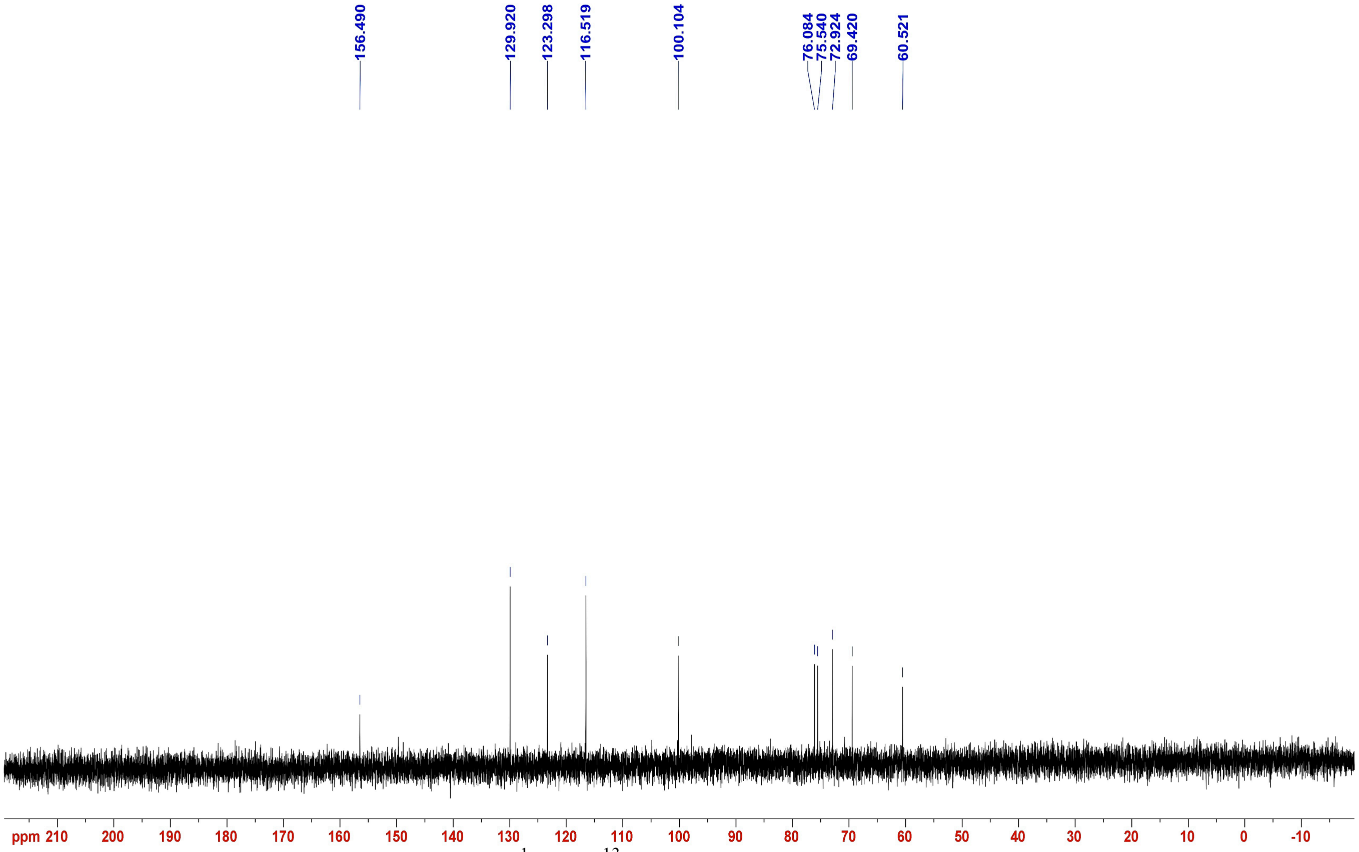

Figure S15. ^{1}H and ^{13}C spectra of β-pNO_2Ph-Glc (**11**).

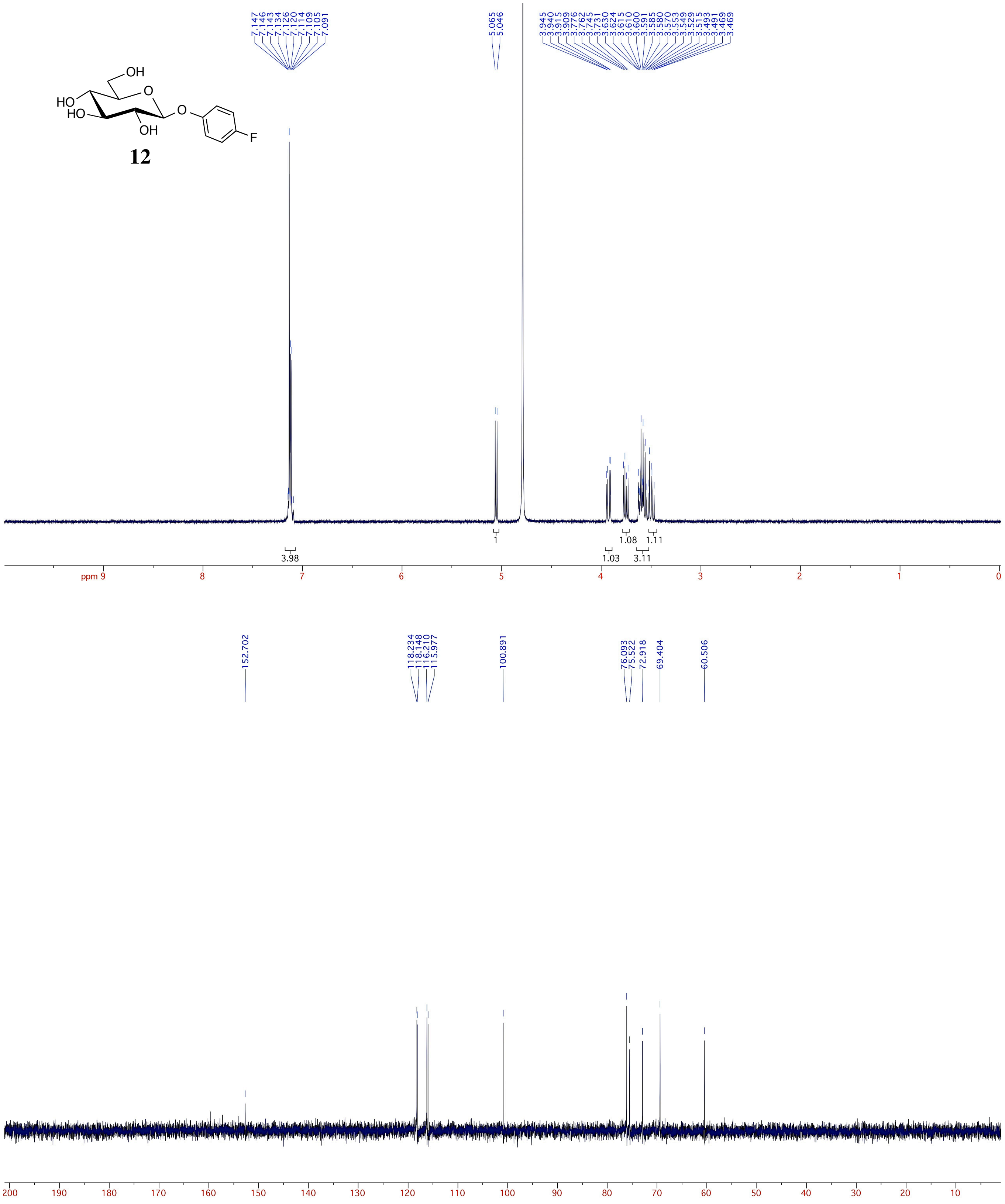

Figure S16. ^{1}H and ^{13}C spectra of β-pFPh-Glc (**12**).

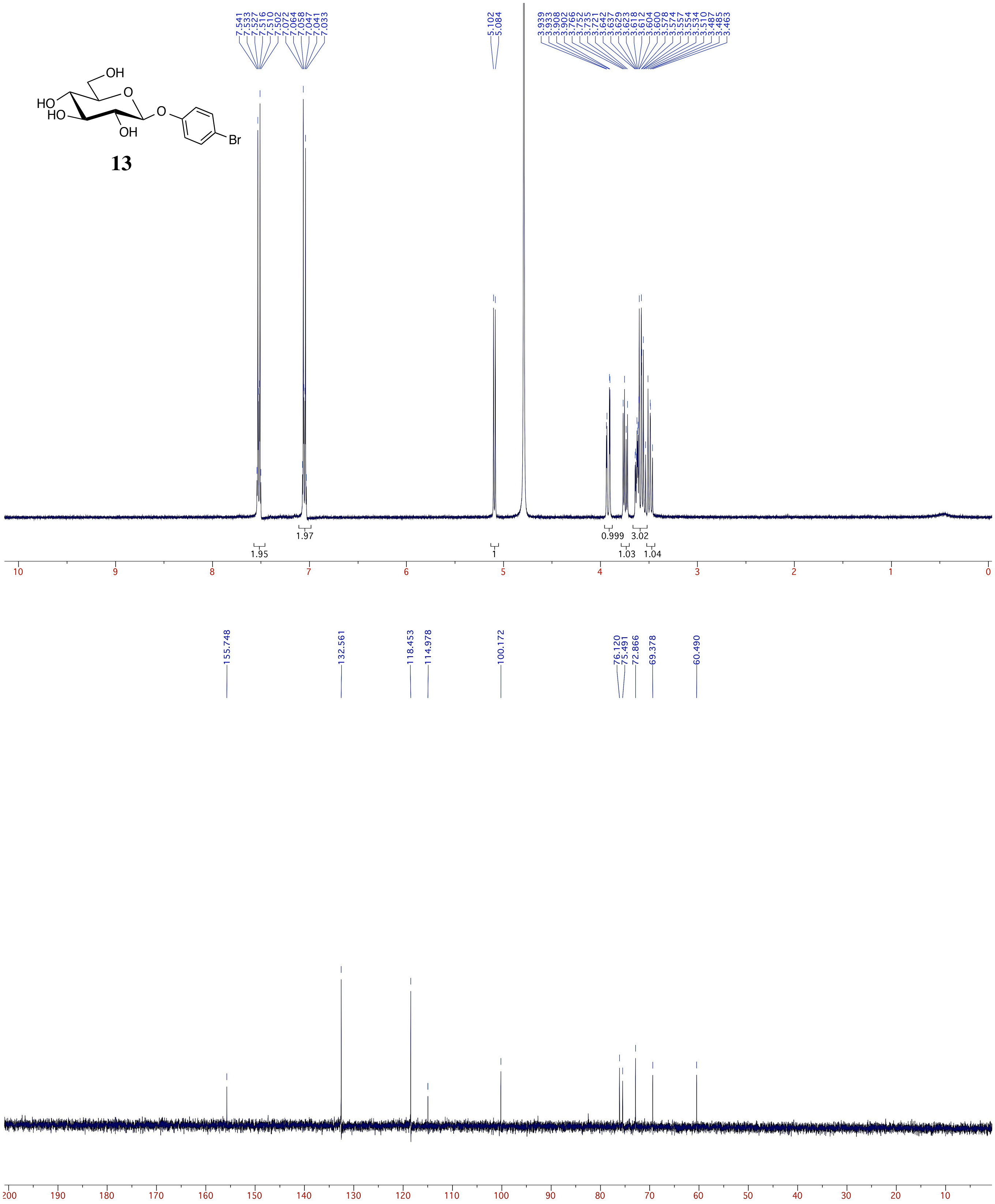

Figure S17. ^{1}H and ^{13}C spectra of β-pBrPh-Glc (**13**).

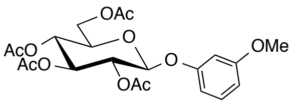

14a

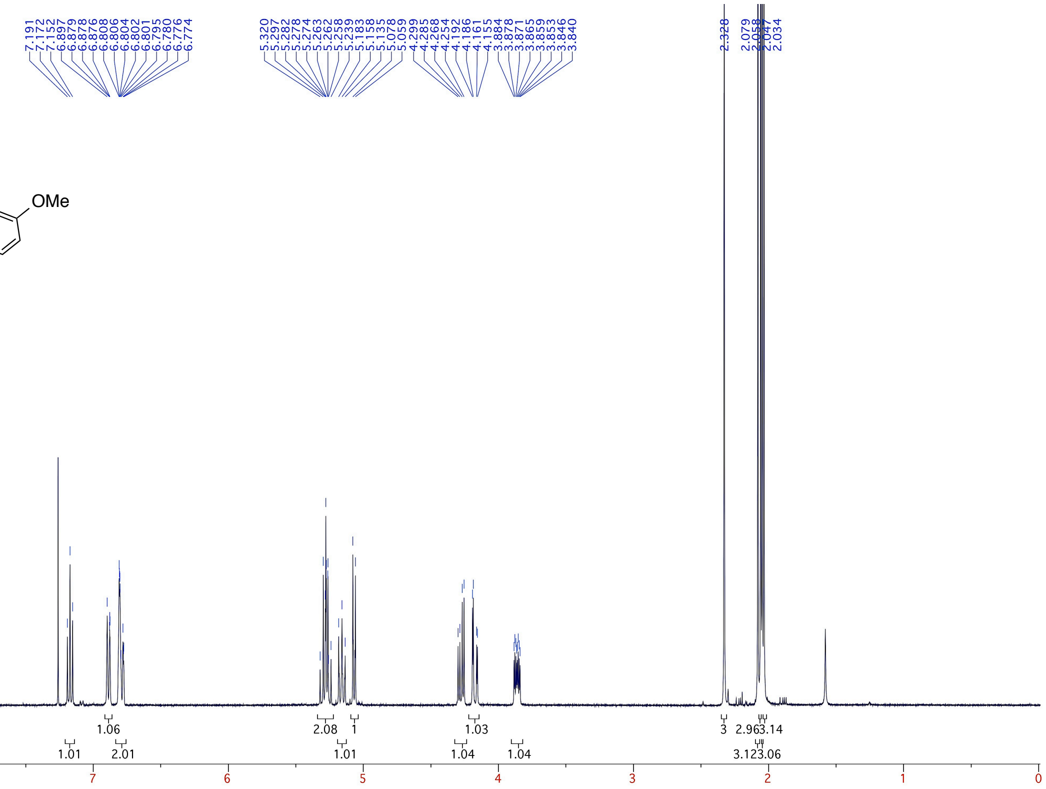

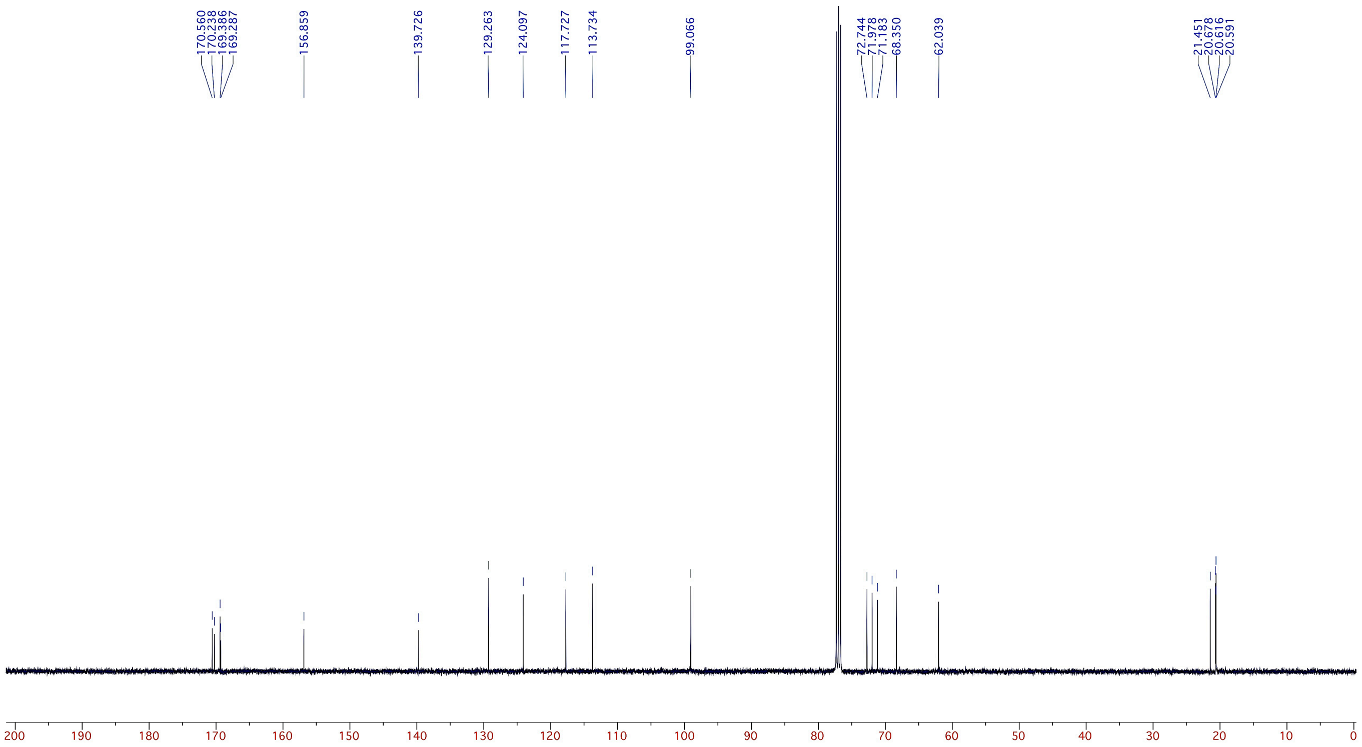

Figure S18. ^{1}H and ^{13}C spectra of **14a**.

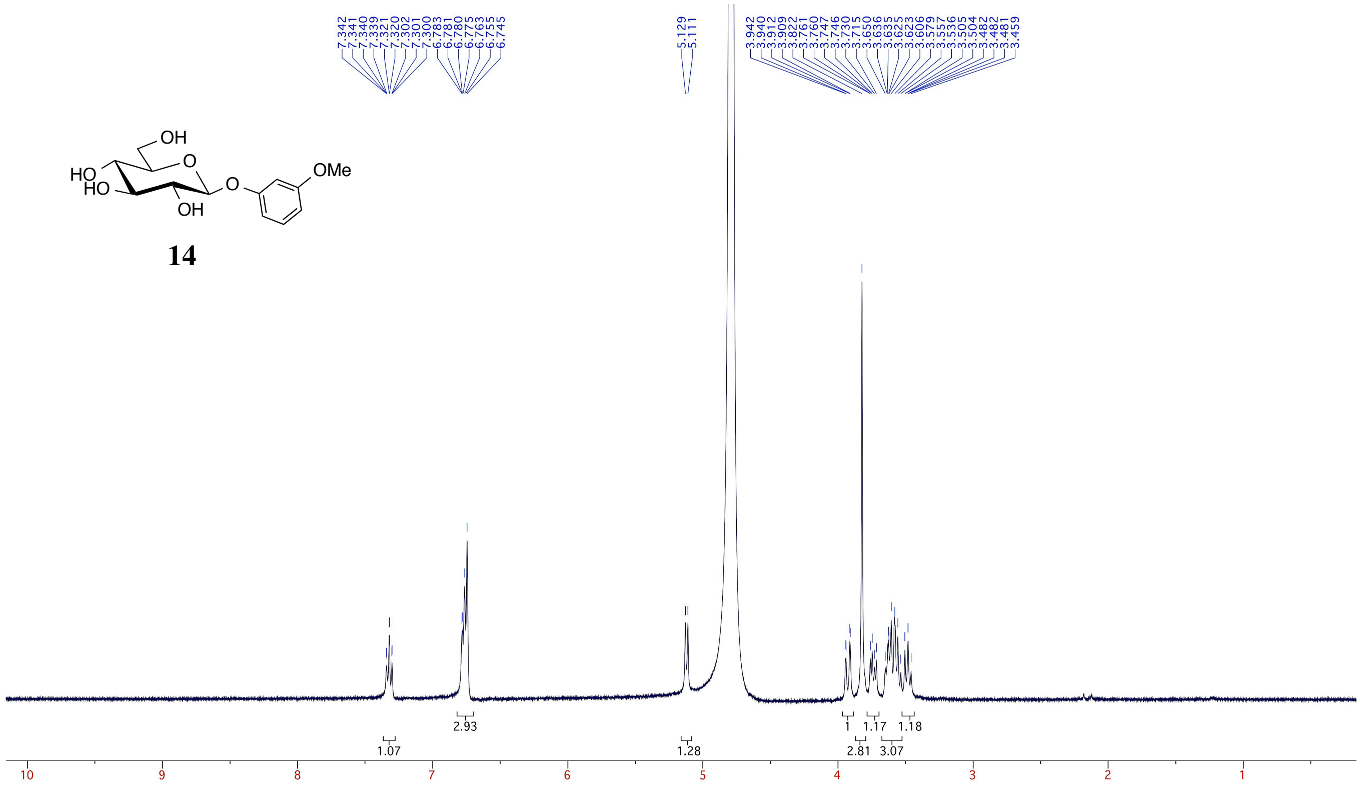

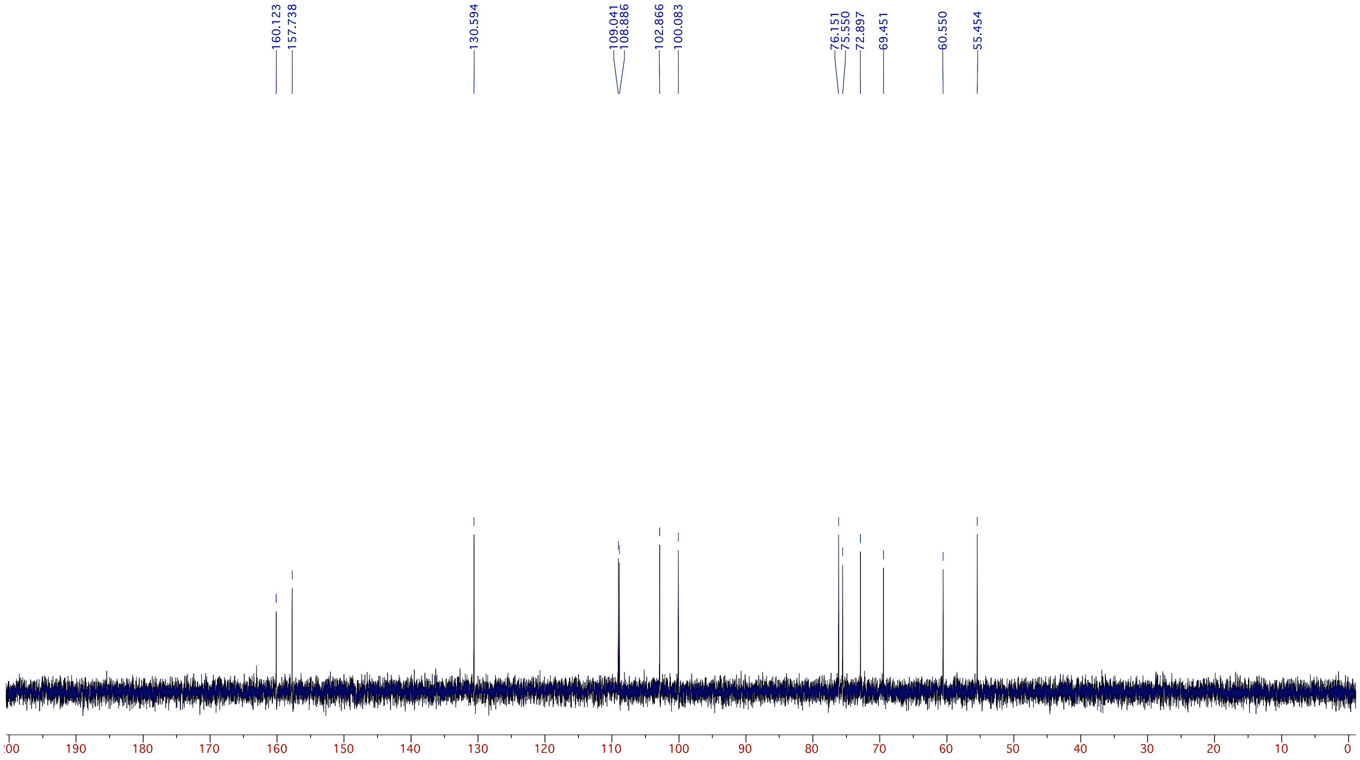

Figure S19. ^{1}H and ^{13}C spectra of β-mOMePh-Glc (**14**).

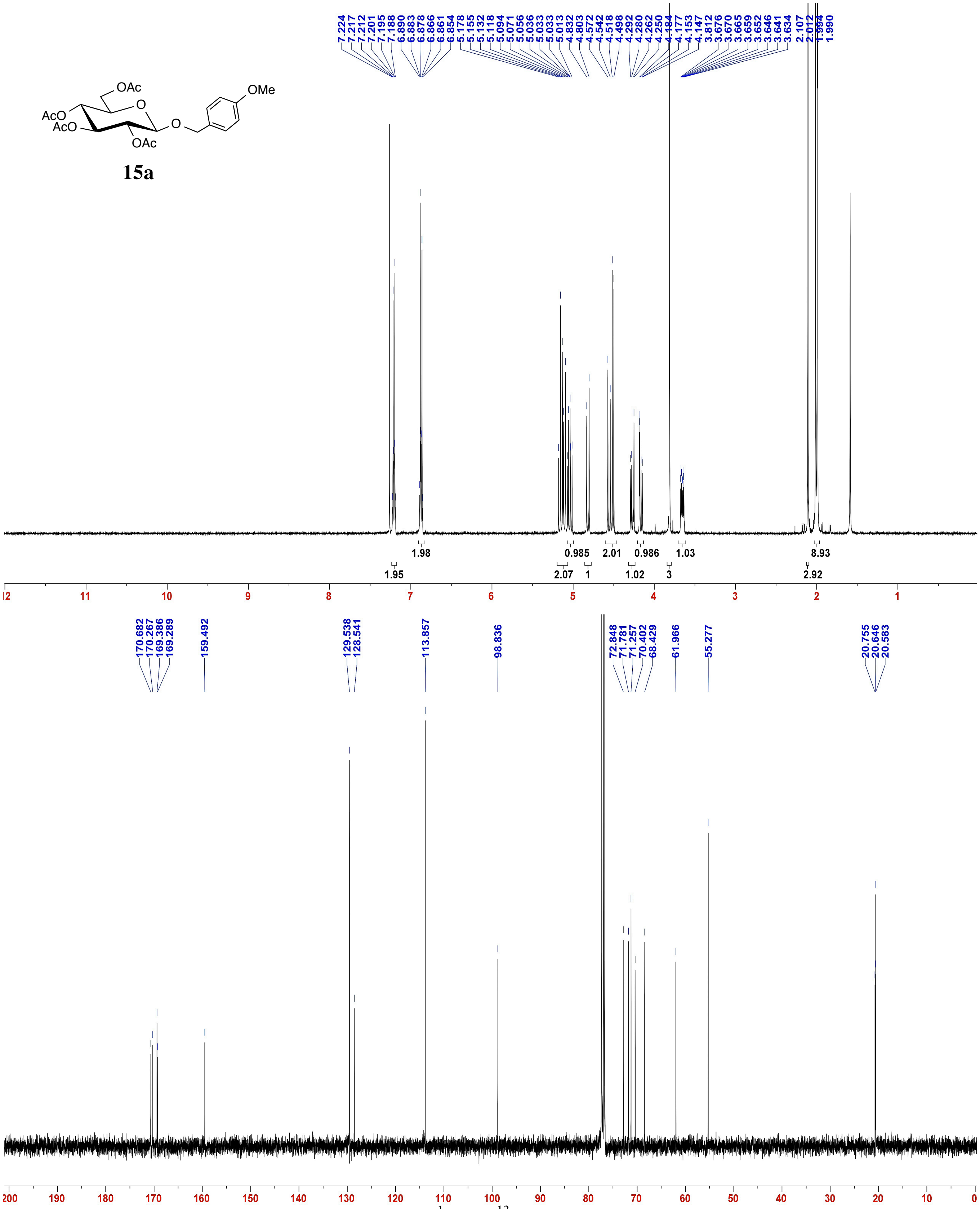

Figure S20. ^{1}H and ^{13}C spectra of **15a**.

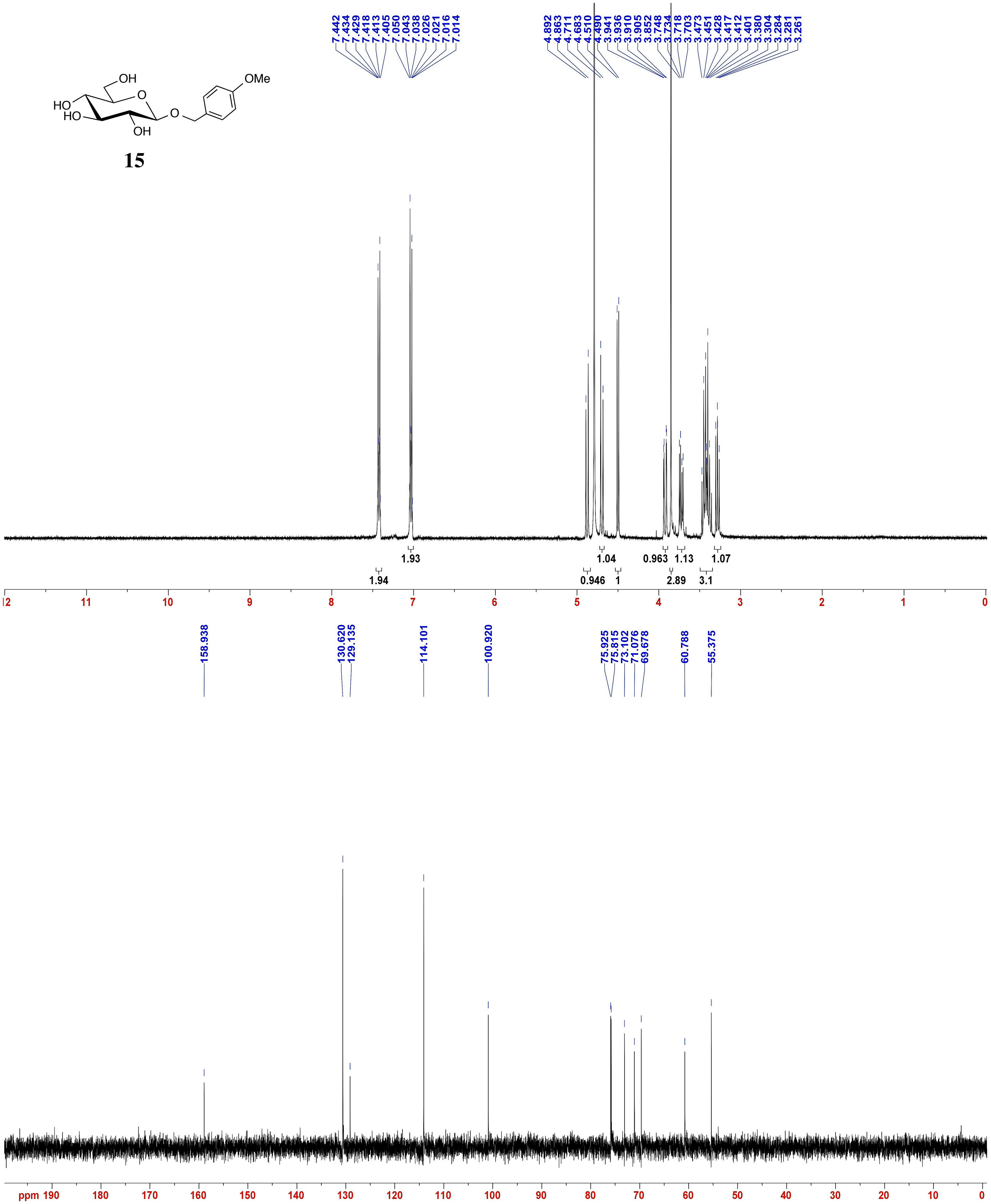

Figure S21. ^{1}H and ^{13}C spectra of β-PMB-Glc (**15**).

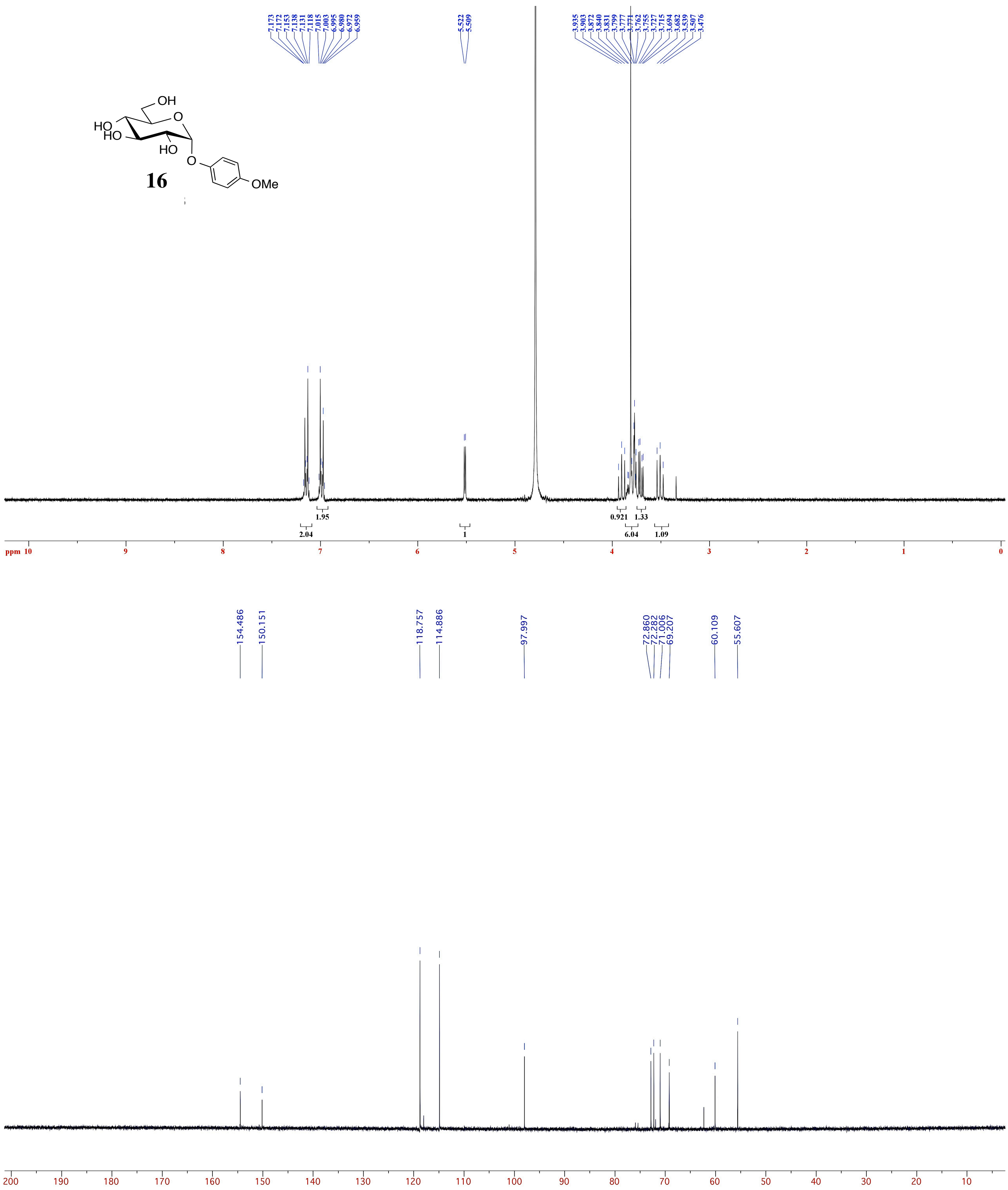

Figure S22. ^{1}H and ^{13}C spectra of α-PMP-Glc (**16**).
